# Association Between Default Number of Opioid Doses in Electronic Health Record Systems and Opioid Prescribing to Adolescents and Young Adults Undergoing Tonsillectomy

**DOI:** 10.1001/jamanetworkopen.2022.19701

**Published:** 2022-06-30

**Authors:** Kao-Ping Chua, Marc C. Thorne, Sophia Ng, Mary Donahue, Chad M. Brummett

**Affiliations:** 1 Department of Pediatrics, Susan B. Meister Child Health Evaluation and Research Center, University of Michigan Medical School, Ann Arbor; 2 Department of Health Management and Policy, University of Michigan School of Public Health, Ann Arbor; 3 Division of Pediatric Otolaryngology, Department of Otolaryngology, University of Michigan Medical School, Ann Arbor; 4 Michigan Opioid Prescribing Engagement Network, University of Michigan Medical School, Ann Arbor; 5 Division of Pain Medicine, Department of Anesthesiology, University of Michigan Medical School, Ann Arbor

## Abstract

**Importance:**

In prior studies, decreasing the default number of doses in opioid prescriptions written in electronic health record systems reduced opioid prescribing. However, these studies did not rigorously assess patient-reported outcomes, and few included pediatric patients.

**Objective:**

To evaluate the association between decreasing the default number of doses in opioid prescriptions written in electronic health record systems and opioid prescribing and patient-reported outcomes among adolescents and young adults undergoing tonsillectomy.

**Design, Setting, and Participants:**

This nonrandomized clinical trial included adolescents and young adults aged 12 to 50 years undergoing tonsillectomy from October 1, 2019, through July 31, 2021, at a tertiary medical center. The treatment group comprised patients from a pediatric otolaryngology service (mostly aged 12-21 years) and the control group comprised patients from a general otolaryngology service (mostly aged 18-25 years).

**Interventions:**

Data on patient-reported opioid consumption and outcomes were collected via a survey on postoperative day 14. Based on opioid consumption among pediatric otolaryngology patients before the intervention, the default number of opioid doses was decreased from 30 to 12 in a tonsillectomy order set. This change occurred only for pediatric otolaryngology patients.

**Main Outcomes and Measures:**

Proportion of patients with 12 doses in the discharge opioid prescription, number of doses in this prescription, and refills and pain-related visits within 2 weeks of surgery. In a secondary analysis of patients completing the postoperative survey, patient-reported opioid consumption, pain control, sleep disturbance, anxiety, and depression were assessed. Linear or log-linear difference-in-differences models were fitted, adjusting for patients’ demographic characteristics and presence of a mental health or substance use disorder.

**Results:**

The study included 237 patients (147 female patients [62.0%]; mean [SD] age, 17.3 [3.6] years). Among 131 pediatric otolaryngology patients, 1 of 70 (1.4%) in the preintervention period and 27 of 61 (44.3%) in the postintervention period had 12 doses in the discharge opioid prescription (differential change, 45.5 percentage points; 95% CI, 32.2-58.8 percentage points). Among pediatric otolaryngology patients, the mean (SD) number of doses prescribed in the preintervention period was 22.3 (7.4) and in the postintervention period was 16.1 (6.5) (differential percentage change, –29.2%; 95% CI, –43.2% to –11.7%). The intervention was not associated with changes in refills or pain-related visits. The secondary analysis included 150 patients. The intervention was not associated with changes in patient-reported outcomes except for a 3.5-point (95% CI, 1.5-5.5 points) differential increase in a sleep disturbance score that ranged from 4 to 20, with higher scores indicating poorer sleep quality.

**Conclusions and Relevance:**

This nonrandomized clinical trial suggests that evidence-based default dosing settings may decrease perioperative opioid prescribing among adolescents and young adults undergoing tonsillectomy, without compromising analgesia.

**Trial Registration:**

ClinicalTrials.gov Identifier: NCT04066829

## Key Points

**Question:**

Is decreasing the default number of doses in opioid prescriptions written in electronic health record systems associated with prescribing or patient-reported outcomes for adolescents and young adults undergoing tonsillectomy?

**Findings:**

In this nonrandomized clinical trial including 237 patients, the default number of doses in opioid prescriptions was lowered from 30 to 12, a level based on prospectively collected data on patient-reported opioid consumption. Compared with controls, this intervention was associated with a significant 46–percentage point increase in the proportion of opioid prescriptions with 12 doses but no changes in pain control.

**Meaning:**

This study suggests that evidence-based default settings may reduce perioperative opioid prescribing without compromising analgesia.

## Teaser

This nonrandomized clinical trial evaluates the association of decreasing the default number of doses in opioid prescriptions written in electronic health record (HER) systems with opioid prescribing and patient-reported outcomes among adolescents and young adults undergoing tonsillectomy.

## Introduction

Opioid prescribing to adolescents and young adults increases their risk of prescription opioid misuse, a risk factor for opioid use disorder.^1,2^ Leftover opioids from prior prescriptions are a key driver of prescription opioid misuse.^3^ Because surgery is one of the most common indications for opioid prescriptions among adolescents and young adults, ensuring that quantities in surgical opioid prescriptions match patient need is crucial.^4^

One approach to achieve this goal is to develop procedure-specific opioid prescribing guidelines based on patient-reported opioid consumption.^5^ Although these guidelines can reduce opioid prescribing, their effectiveness is limited if clinicians are unaware of them.^6,7,8,9,10^ An alternative approach that does not depend on clinician awareness is to implement evidence-based default dosing settings for opioid prescriptions written in electronic health record systems. This behavioral intervention could “nudge” clinicians to prescribe opioid quantities that match patient need by implicitly signaling the recommended behavior and making it easy to choose this behavior.^11^ At the same time, this intervention preserves clinician autonomy by allowing them to prescribe a lower or higher number of doses when indicated.

Several studies suggest that implementing default dosing settings or lowering preexisting settings for opioid prescriptions can reduce opioid prescribing in several clinical settings, including surgery.^12,13,14,15,16,17,18,19,20^ Although important, these studies had limitations. First, most either failed to measure patient-reported outcomes or measured only a limited number of these outcomes, precluding rigorous assessment for unintended effects. Second, most used an uncontrolled pre-post design.^14,17^ Third, few included pediatric patients. Fourth, few used default dosing settings that were based on data on patient-reported opioid consumption, an indicator of opioid need. For example, several studies evaluated the effect of implementing institution-wide default settings that were uniform across all conditions.^14,17^

In this study, we prospectively collected patient-reported opioid consumption data among adolescents and young adults undergoing tonsillectomy, a common procedure for which opioids are frequently prescribed.^21,22,23,24^ We subsequently assessed changes in opioid prescribing after we lowered the default number of doses in discharge opioid prescriptions to a level that accounts for the needs of most patients. To maximize rigor, we used a difference-in-differences approach and assessed whether the intervention was associated with changes in refills, pain-related return visits, and a robust set of patient-reported outcomes.

## Methods

### Study Participants

Participants were adolescents and young adults aged 12 to 25 years undergoing palatine tonsillectomy with or without adenoidectomy at the University of Michigan from October 1, 2019, through March 31, 2020, or from June 1, 2020, through July 31, 2021. The study period excluded April and May 2020, when elective procedures at our institution were suspended owing to COVID-19 (trial protocol in Supplement 1). eAppendix 1 in Supplement 2 lists other pandemic-related protocol changes. This study was approved by the institutional review board of the University of Michigan Medical School. We obtained written or verbal consent from young adults aged 18 to 25 years, depending on recruitment method. For adolescents aged 12 to 17 years, a parent provided written or verbal consent, while the adolescent provided verbal assent. This study followed the Transparent Reporting of Evaluations With Nonrandomized Designs (TREND) reporting guideline.

At our institution, the pediatric otolaryngology service cares predominantly for patients aged 21 years or younger. The general otolaryngology service cares predominantly for patients aged 18 years or older. Patients were eligible if they underwent tonsillectomy performed by either a pediatric otolaryngology attending physician at C. S. Mott Children’s Hospital or by a general otolaryngology attending physician at a site other than this hospital, and if they were not any of the following: patients living in foster care or with a legal guardian, patients with medical complexity or developmental delays, non–English-speaking patients, patients with recent suicidal ideation documented in their medical record, or patients undergoing emergency tonsillectomy.

We recruited eligible patients either by telephone during the 2 weeks before surgery or in person on the day of surgery. By enrolling, patients granted the study team permission to examine their electronic health record data and to send them 3 surveys: a baseline survey assessing prior opioid use and patient-reported outcomes (depression, anxiety, and sleep disturbance); daily surveys assessing opioid consumption and pain control on postoperative days 1 to 13; and a survey on postoperative day 14 assessing patient-reported outcomes and opioid consumption. We chose to survey patients on postoperative day 14 rather than later to facilitate recall of these outcomes. Patients received a $10 gift card per survey completed. Surveys were administered online via Qualtrics, except for patients recruited in person, who completed the baseline survey on tablets.

The study involved 2 analyses, each with different samples. The primary analysis focused on outcomes contained in our Epic-based electronic health record system, such as refills. Eligible patients were included unless they were reached for recruitment but declined to enroll, enrolled but later withdrew from participation or ultimately did not undergo tonsillectomy, were not prescribed opioids at discharge, had opioid use in the 90 days before surgery, were enrolled in another study, were hospitalized for more than 1 day after surgery, underwent another major procedure at the same time as tonsillectomy, or opted for an opioid-sparing pathway (eAppendix 2 in Supplement 2). To increase sample size in light of decreased tonsillectomy volume during the COVID-19 pandemic, we received permission from our institutional review board to include patients who could not be reached for recruitment in the primary analysis.

The secondary analysis focused on patient-reported outcomes measured in the 2-week postoperative survey. Among patients in the primary analysis, we excluded those who did not complete both the baseline and 2-week surveys. We did not exclude patients who failed to complete the daily surveys, the results of which will be reported in a future article.

### Study Design

We used a difference-in-differences design. In this design, the difference between outcomes during the preintervention and postintervention periods in the control group is subtracted from the corresponding difference in the treatment group. The resulting differential change represents the intervention’s effect, assuming that outcome trends in the treatment group would have been similar to those in the control group without the intervention. Confidence in this assumption is increased if preintervention trends between groups are parallel.^25,26^ The treatment group comprised patients in the pediatric otolaryngology service and the control group comprised patients in the general otolaryngology service. The preintervention period was from October 1, 2019, to September 30, 2020 (excluding April and May 2020). The postintervention period was from October 1, 2020, to July 31, 2021. eAppendix 3 in Supplement 2 depicts the study design.

### Intervention

At our institution, residents and attending physicians on the pediatric otolaryngology service typically use a tonsillectomy discharge order set that includes orders for opioid prescriptions, ibuprofen, and acetaminophen. Clinicians must search for this order set manually, although many add it to their list of favorite orders. Before the intervention, the order set had a single pathway for patients aged 12 years or older. The default opioid prescription was oxycodone liquid (5 mg/5 mL) at 0.1 mg/kg per dose (maximum, 5 mg per dose). The default number of doses was 30, the division standard.

The intervention involved 2 changes to the order set. First, we split the pathway for patients aged 12 years or older into 3 pathways: oxycodone liquid, 0.1 mg/kg per dose for patients weighing less than 50 kg (maximum, 5 mg per dose); oxycodone liquid, 5 mg per dose for patients weighing 50 kg or more; and oxycodone tablet, 5 mg per dose for patients weighing 50 kg or more. Second, in all pathways, we decreased the default number of doses in discharge opioid prescriptions from 30 to 12. We chose 12 doses after reviewing opioid consumption data reported by pediatric otolaryngology patients undergoing tonsillectomy from October 1, 2019, through July 31, 2020. Median opioid consumption was 6.5 doses (25th-75th percentile, 2.3-14.2 doses), suggesting that 12 doses would cover the needs of most patients.

The order set changed only at C. S. Mott Children’s Hospital. If clinicians used this order set elsewhere, the older version displayed. Because we wanted to understand whether changing default settings can decrease opioid prescribing without awareness of the change, the chief of pediatric otolaryngology (M.C.T.) sent an email to attending physicians and residents announcing that the order set was changing without specifying further. Other clinicians were blinded to the intervention, as were patients. The chief of pediatric otolaryngology was the attending physician for only 5 study patients. eAppendix 4 in Supplement 2 includes additional details on the intervention, including screenshots of the order set.

### Outcomes

All outcomes were patient-level outcomes. In the primary analysis, we calculated 5 outcomes: the proportion of patients with 12 doses in the discharge opioid prescription (to assess adherence to the new defaults), mean number of doses in the discharge opioid prescription, and the proportion of patients with the following during the 2 weeks after surgery: 1 or more refills from a University of Michigan clinician, 1 or more office visits at the University of Michigan because of pain, and 1 or more emergency department visits or hospitalizations at the University of Michigan because of pain.

The secondary analysis had 18 outcomes, including opioid consumption during the 2 weeks after surgery, patient satisfaction with pain control, having well-controlled pain, having pain that was worse or much worse than expected, level of pain at its worst and on average during the past 7 days, presence and disposal of leftover opioids, opioid misuse, depression, anxiety, and sleep disturbance. We also assessed visits for poorly controlled pain during the 2 weeks after surgery regardless of whether visits occurred at the University of Michigan, including primary care visits, retail clinic visits, urgent care center visits, emergency department visits, and hospitalizations. When possible, we used validated measures to assess patient-reported outcomes, such as the Patient-Reported Outcomes Measurement Information System (PROMIS) measures for anxiety and sleep disturbance as well as the 8-item Patient Health Questionnaire (PHQ-8), a depression screening tool.^27^ eAppendix 5 in Supplement 2 includes the 2-week survey instrument and eAppendix 6 in Supplement 2 describes the calculation of outcomes in the secondary analysis.

### Statistical Analysis

We used linear difference-in-differences models for all outcomes except for the number of doses prescribed, for which we used a log-linear model owing to the nonnormal distribution and better model fit. In all models, we used the improved doubly robust difference-in-differences estimator, which combines outcome regression adjustment with inverse probability weighting to reduce bias.^28^ Models adjusted for several covariates derived from the electronic health record, including sex, insurance type, presence of a mental health or substance use disorder, and self-reported race and ethnicity, which was included as a covariate to account for potential racial and ethnic disparities in postsurgical pain management. For models assessing scores on the PHQ-8 and the 2 PROMIS measures for anxiety and sleep disturbance, we also controlled for patients’ scores from the baseline survey. For the secondary analysis, we excluded patients from individual analyses if they had missing data for the outcome or any covariate. No patients in the primary analysis had missing data.

To evaluate the parallel trends assumption, we subset to data from the preintervention period and fitted linear or log-linear models with terms for month, treatment group status, their interaction (the coefficient of interest), and the same covariates. All models used bootstrapped standard errors with 1000 iterations. To assess statistical power, we examined the width of 95% CIs. We conducted analyses using the DRDID package in R, version 4.0.3 (R Group for Statistical Computing) and 2-sided hypothesis tests with α = .05.

## Results

### Study Cohort

**Figure 1.**
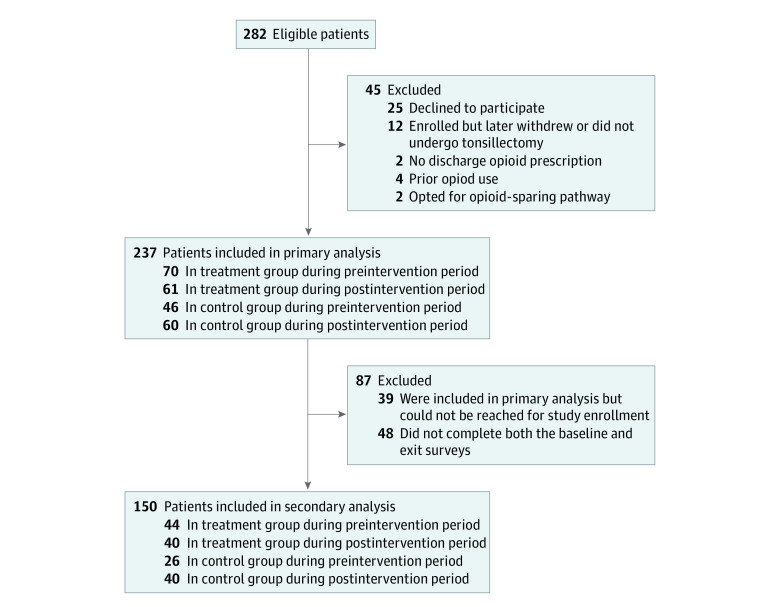
Sample Inclusion and Exclusion Criteria for Primary and Secondary Analyses A total of 318 patients were screened for eligibility, of whom 282 met eligibility requirements. No patients were excluded from the primary analysis because they were enrolled in another study, were hospitalized for more than 1 day after surgery, or underwent another major procedure at the same time as tonsillectomy. The primary analysis included 39 eligible patients who could not be reached for enrollment to complete the surveys. Because these patients did not complete surveys, they were not included in the secondary analysis. In analyses of each of the 18 outcomes in the secondary analysis, patients with missing data for the outcome were excluded (eTable 3 in Supplement 2).

Figure 1 shows the number of patients included and excluded from the primary and secondary analyses. Among 282 eligible patients, 45 (16.0%) were excluded from the primary analysis, leaving 237 patients (147 female patients [62.0%]; mean [SD] age, 17.3 [3.6] years). Of these, 87 (36.7%) were excluded from the secondary analysis, leaving 150 patients.

Table 1 shows characteristics of the patients in the primary analysis by treatment group. eTable 1 in Supplement 2 displays characteristics by treatment group and postintervention status. Of the 237 patients included, 131 (55.3%) were pediatric otolaryngology patients, of whom 119 (90.8%) were aged 12 to 17 years, 74 (56.5%) were female, 98 (74.8%) were White non-Hispanic, and 93 (71.0%) were privately insured. Fewer pediatric otolaryngology patients were aged 18 to 25 years than general otolaryngology patients (12 of 131 [9.2%] vs 98 of 106 [92.5%]).

**Table 1.** Characteristics of Patients Included in the Primary Analysis

Characteristic	Pediatric otolaryngology, No. (%) (n = 131)	General otolaryngology, No. (%) (n = 106)	*P* value^a^	Standardized mean difference
Surgery date				
October to November 2019	14 (10.7)	5 (4.7)	.78	0.31
December 2019 to January 2020	18 (13.7)	16 (15.1)		
February to March 2020	10 (7.6)	6 (5.7)		
June to July 2020	16 (12.2)	10 (9.4)		
August to September 2020	12 (9.2)	9 (8.5)		
October to November 2020	5 (3.8)	6 (5.7)		
December 2020 to January 2021	13 (9.9)	15 (14.2)		
February to March 2021	11 (8.4)	11 (10.4)		
April to May 2021	12 (9.2)	13 (12.3)		
June to July 2021	20 (15.3)	15 (14.2)		
Age group, y				
12-17	119 (90.8)	8 (7.5)	<.001	3.01
18-25	12 (9.2)	98 (92.5)		
Sex				
Male	55 (42.0)	32 (30.2)	.12	0.26
Female	74 (56.5)	73 (68.9)		
Other^b^	2 (1.5)	1 (0.9)		
Race and ethnicity^c^				
Asian, Non-Hispanic	4 (3.1)	3 (2.8)	.99	0.10
Black, Non-Hispanic	12 (9.2)	9 (8.5)		
Hispanic, any race	9 (6.9)	6 (5.7)		
White, Non-Hispanic	98 (74.8)	83 (78.3)		
Other, multiracial, non-Hispanic	4 (3.1)	3 (2.8)		
Unknown race and/or ethnicity	4 (3.1)	2 (1.9)		
Payer type				
Private	93 (71.0)	85 (80.2)	.13	0.22
Medicaid	38 (29.0)	21 (19.8)		
Mental health or substance use disorders				
Yes	63 (48.1)	47 (44.3)	.60	0.08
No	68 (51.9)	59 (55.7)		
Indication for tonsillectomy				
Sleep-disordered breathing, with or without tonsillitis	92 (70.2)	10 (9.4)	<.001	1.62
Tonsillitis without sleep-disordered breathing	32 (24.4)	89 (84.0)		
Other	7 (5.3)	7 (6.6)		
Procedure type				
Tonsillectomy alone	32 (24.4)	92 (86.8)	<.001	1.61
Tonsillectomy with adenoidectomy	99 (75.6)	14 (13.2)		
Resident involved in surgery				
Yes	85 (64.9)	47 (44.3)	.001	0.42
No	46 (35.1)	59 (55.7)		
Hospitalized after surgery				
Yes	24 (18.3)	2 (1.9)	<.001	0.57
No	107 (81.7)	104 (98.1)		

^a^ Derived from the Fisher exact test.

^b^ Other included categories other than male or female, such as nonbinary. These data were derived from the electronic health record and are based on self-report.

^c^ Data were derived from the electronic health record and are based on self-report.

eTable 2 in Supplement 2 displays characteristics of patients in the secondary analysis by treatment group. Of the 150 patients included, 84 (56.0%) were on the pediatric otolaryngology service. The number of patients excluded owing to missing data was 4 or less for 16 of the 18 analyses, 11 for 1 analysis, and 15 for 1 analysis (eTable 3 in Supplement 2).

#### Primary Analysis

**Figure 2.**
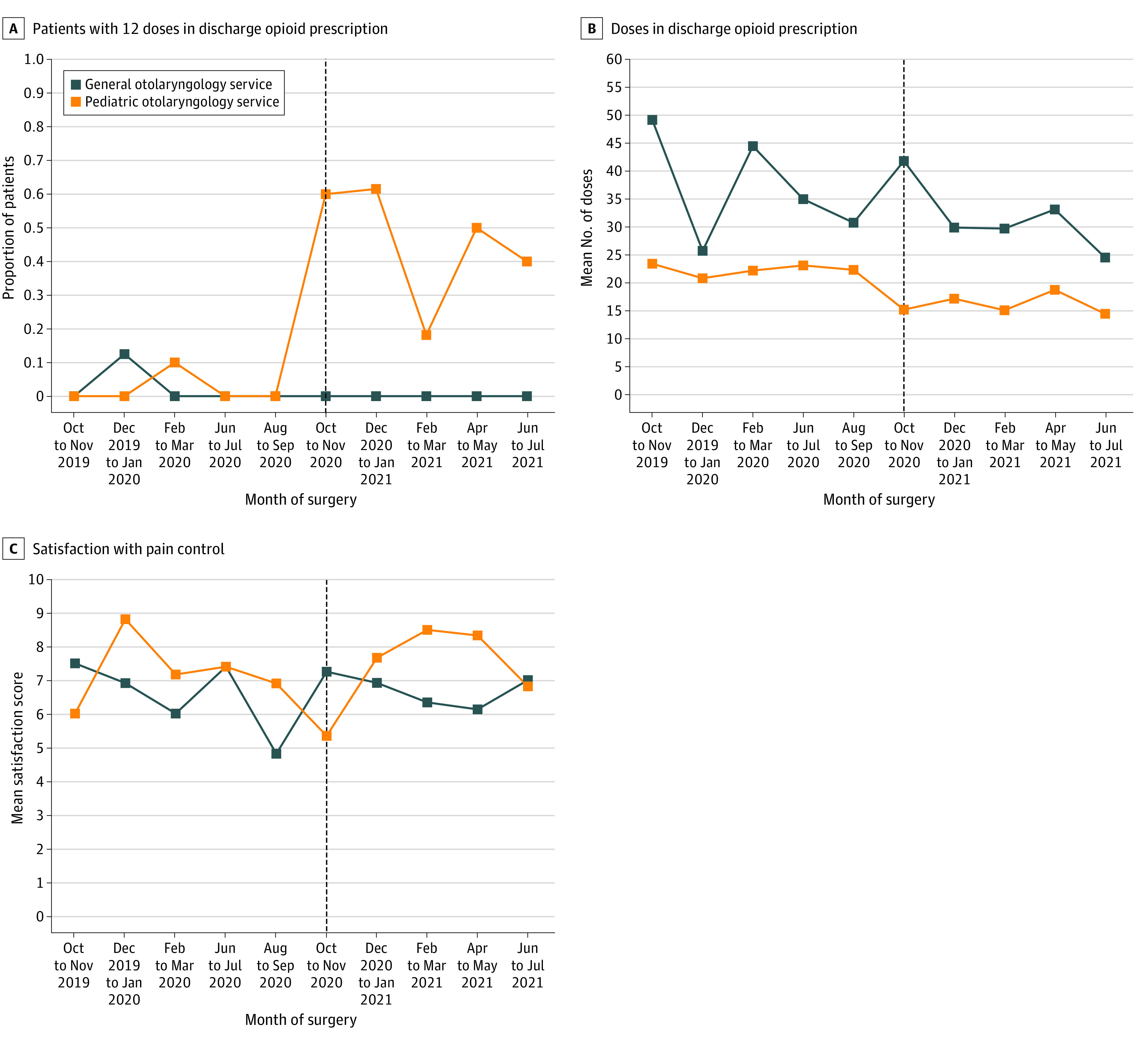
Selected Outcomes in the Pediatric Otolaryngology vs General Otolaryngology Service A, Proportion of patients in the primary analysis with 12 doses in the discharge opioid prescription. B, Mean number of doses in the discharge opioid prescription in the primary analysis. C, Mean satisfaction with pain control in the secondary analysis. Outcomes are aggregated to bimonthly periods. The vertical dashed line denotes October 2020, the month during which the default number of doses was decreased from 30 to 12 in a tonsillectomy order set. This change occurred only for the pediatric otolaryngology service. The months of April and May 2020 are not displayed; during these months, elective procedures such as tonsillectomy were suspended at our institution owing to COVID-19.

Table 2 displays results from the primary analysis. Among 131 pediatric otolaryngology patients, 1 of 70 (1.4%) in the preintervention period and 27 of 61 (44.3%) in the postintervention period had 12 doses in the discharge opioid prescription. Among 106 general otolaryngology patients, 2 of 46 (4.3%) in the preintervention period and no patients in the postintervention period had 12 doses in this prescription (differential change, 45.5 percentage points; 95% CI, 32.2-58.8 percentage points; Figure 2). Among pediatric otolaryngology patients, the mean (SD) number of doses prescribed was 22.3 (7.4) in the preintervention period and 16.1 (6.5) in the postintervention period, compared with 33.7 (20.4) in the preintervention period and 30.4 (12.4) in the postintervention period for general otolaryngology patients (differential percentage change, –29.2%; 95% CI, –43.2% to –11.8%). There was no differential change in refill rates (22.6 percentage points; 95% CI, –0.4 to 45.5 percentage points) or visits for pain at the University of Michigan during the 2 weeks after surgery. Preintervention trends were similar between groups for all outcomes.

**Table 2.** Primary Opioid-Related Outcomes Among Patients Receiving Prescriptions Before vs After Intervention

Outcome	Pediatric otolaryngology service, No. (%) (n = 131)		General otolaryngology service, No. (%) (n = 106)		Adjusted difference-in-differences estimate, coefficient (95% CI)
	Preintervention period (n = 70)	Postintervention period (n = 61)	Preintervention period (n = 46)	Postintervention period (n = 60)	
Patients with discharge opioid prescriptions for 12 doses	1 (1.4)	27 (44.3)	2 (4.3)	0	45.5 (32.2 to 58.8)
Doses in discharge opioid prescriptions, mean (SD)^a^	22.3 (7.4)	16.1 (6.5)	33.7 (20.4)	30.4 (12.4)	–29.2^b^ (–43.2 to –11.8)
Patients who had at least one refill from a University of Michigan clinician during the 2 wk after surgery	10 (14.3)	12 (19.7)	25 (54.3)	26 (43.3)	22.6 (–0.4 to 45.5)
Patients who had an office visit with a University of Michigan clinician because of pain during the 2 wk after surgery^c^	2 (2.9)	1 (1.6)	0	1 (1.7)	–2.4 (–8.02 to 3.3)
Patients who had an emergency department visit or hospitalization at the University of Michigan because of pain during the 2 wk after surgery^c^	3 (4.3)	2 (3.3)	2 (4.4)	3 (5.0)	1.0 (–8.52 to 1.1)

^a^ For patients hospitalized after surgery, the discharge opioid prescription was the prescription written on the day of hospital discharge. In the primary and secondary analyses, all patients had discharge opioid prescriptions. Only 2 patients were excluded owing to lack of a discharge opioid prescription.

^b^ Unlike the other 4 outcomes, we modeled the log of the number of doses in discharge opioid prescriptions owing to nonnormal distribution of residuals and better model fit (based on the Akaike Information Criterion). The difference-in-differences estimate represents the additional percentage change in the number of doses prescribed on the pediatric otolaryngology service compared with the counterfactual value (ie, the number of doses that would have been prescribed on the pediatric otolaryngology service had it experienced the same percentage change in doses prescribed as the general otolaryngology service).

^c^ We considered office visits, emergency department visits, or hospitalizations because of dehydration to be pain related.

Among 61 pediatric otolaryngology patients in the postintervention period, 52 (85.2%) were prescribed opioids through the tonsillectomy order set, of whom 27 (51.9%) had 12 doses in the discharge prescription. Among the 9 patients who were not prescribed opioids through this order set, none had 12 doses in the discharge prescription. Among the 61 pediatric otolaryngology patients in the postintervention period, the proportion prescribed 12 doses did not vary by patient characteristics or whether prescribers were attending physicians vs residents (eTable 4 in Supplement 2).

#### Secondary Analysis

With only one exception, the intervention was not associated with changes in outcomes in the secondary analysis (Table 3). For example, among pediatric otolaryngology patients, the mean (SD) satisfaction with pain control was 7.4 (2.4) in the preintervention period and 7.4 (2.4) in the postintervention period (differential change, –0.5; 95% CI, –2.0 to 1.1). The exception was a differential increase in the PROMIS sleep disturbance score, which ranges from 4 to 20, with higher scores being worse. Among pediatric otolaryngology patients, the mean sleep disturbance score increased by 1.3 points from the preintervention to the postintervention period, compared with a 1.0-point decrease among patients in the general otolaryngology service (differential change, 3.5 points; 95% CI, 1.5-5.5 points). Preintervention trends were similar between groups for all outcomes.

**Table 3.** Results of Secondary Analysis

Outcome	Pediatric otolaryngology service, No./total No. (%) (n = 84)^a^		General otolaryngology service, No./total No. (%) (n = 66)^a^		Adjusted difference-in-differences estimate, coefficient (95% CI)
	Preintervention period (n = 44)	Postintervention period (n = 40)	Preintervention period (n = 26)	Postintervention period (n = 40)	
Satisfaction with pain control, mean (SD)	7.4 (2.4)	7.4 (2.4)	6.5 (2.5)	6.7 (7.0)	–0.5 (–2.0 to 1.1)
Patients reporting well-controlled pain (vs adequately or poorly controlled pain)	32/44 (72.7)	25/39 (64.1)	11/25 (44.0)	18/40 (45.0)	–2.2 (–34.4 to 30.0)
Patients reporting pain was worse or much worse than expected (vs about what was expected, better than expected, and much better than expected)	15/44 (34.1)	11/39 (28.2)	11/26 (42.3)	18/40 (45.0)	–1.2 (–33.6 to 31.2)
Patients whose pain had resolved by postoperative day 14	32/44 (72.7)	34/40 (85.0)	17/26 (65.4)	24/40 (60.0)	25.6 (–4.2 to 55.3)
Pain score at its worst during the past 7 d, mean (SD)	5.3 (2.8)	5.4 (3.2)	7.1 (2.0)	6.4 (3.1)	1.2 (–0.5 to 2.9)
Pain score during the past 7 d, mean (SD)	3.3 (2.1)	3.3 (3.0)	4.4 (2.0)	3.9 (2.2)	0.7 (–0.8 to 2.2)
No. of opioid doses consumed during the 2 wk after surgery, mean (SD)	7.2 (8.0)	9.5 (9.6)	27.3 (18.7)	23.3 (23.4)	2.5 (–12.1 to 17.1)
Patients with leftover doses of opioids	35/44 (79.5)	28/38 (73.7)	21/26 (80.8)	24/38 (63.2)	15.0 (–13.4 to 43.4)
Patients with leftover doses who disposed of them	28/34 (82.4)	17/25 (68.0)	13/19 (68.4)	17/23 (73.9)	–20.0 (–47.9 to 8.0)
Patients reporting misuse of opioids belonging to others during the 2 wk after surgery	0	0	0	0	NA
Patients reporting misuse of their own opioids during the 2 wk after surgery	0	0	0	0	NA
Score on PHQ-8, mean (SD)^b^	5.1 (5.6)	5.6 (4.9)	7.4 (4.4)	7.6 (5.1)	–0.4 (–3.1 to 2.2)
Score on PROMIS Pediatric Anxiety–Short Form 8a, mean (SD)^b^	11.2 (4.6)	12.4 (6.1)	15.9 (8.1)	13.4 (7.2)	2.8 (–0.3 to 5.9)
Score on PROMIS Sleep Disturbance–Short Form 4a, mean (SD)^b^	8.4 (2.9)	9.7 (3.7)	12.2 (4.0)	11.2 (4.2)	3.5 (1.5 to 5.5)
Patients who visited their primary care physician because of poorly controlled pain	3/44 (6.8)	0	1/25 (4.0)	0	1.9 (–9.9 to 13.7)
Patients who visited an urgent care center because of poorly controlled pain	1/44 (2.3)	1/40 (2.5)	0	1/40 (2.5)	–2.4 (–10.4 to 5.6)
Patients who visited a retail clinic because of poorly controlled pain	1/44 (2.3)	0	3/25 (12.0)	0	6.7 (–5.6 to 19.1)
Patients who went to an emergency department or were hospitalized because of poorly controlled pain	3/44 (6.8)	0	4/25 (16.0)	3/40 (7.5)	1.6 (–20.4 to 23.6)

Abbreviations: NA, not applicable; PHQ-8: 8-item Patient Health Questionnaire (depression scale); PROMIS, Patient-Reported Outcomes Measurement Information System.

^a^ For all but 2 of the outcomes, the number of respondents with missing data ranged from 0 to 4; those respondents were excluded from analyses of that outcome. For the opioid consumption outcome, 15 respondents were excluded owing to missing data. For the disposal of leftover opioids outcome, 7 respondents were excluded owing to missing data.

^b^ For the PHQ-8 and PROMIS measures, higher scores are worse. The raw score is presented. Conclusions were unchanged when assessing the T score for the PROMIS measures. See eAppendix 5 in Supplement 2 for details on score calculation.

## Discussion

We used data on patient-reported opioid consumption to design a simple, low-cost intervention that reduced the default number of doses in opioid prescriptions to a level that accounts for the needs of most adolescents and young adults undergoing tonsillectomy. Compared with a control group, this intervention was associated with an increase in the proportion of opioid prescriptions with the new default number of doses. In contrast, the intervention was not associated with changes in pain-related visits, opioid consumption, pain control, anxiety, or depression.

In prior studies, implementation of opioid prescribing guidelines based on data on patient-reported opioid consumption decreased perioperative opioid prescribing without worsening patient-reported outcomes or increasing refills.^7,8^ In our study, implementing evidence-based default settings similarly did not worsen most patient-reported outcomes. The one exception was an apparent worsening in sleep disturbance, although the clinical significance of this finding is uncertain, given that the magnitude of the change was modest. The intervention was not associated with a significantly increased rate of refills, although a caveat is that significance might have been achieved with a larger sample size. Overall, findings suggest that the intervention reduced opioid prescribing with minimal unintended outcomes, although not none.

We deliberately did not announce the change in default settings to otolaryngology attending physicians and residents. The fact that opioid prescribing nonetheless changed markedly is consistent with prior research on the importance of order set design and suggests that default settings may have the ability to influence clinician behavior.^11,29^ Although this ability could be leveraged to improve outcomes, poorly set default settings could have the opposite effect. To avoid this possibility, default settings should ideally be based on patient-reported opioid consumption data. Such an evidence-based approach increases the chance that the settings will match the amount of opioids that patients need while also potentially increasing clinicians’ comfort with these settings.

Changing default settings cannot eliminate excessive opioid prescribing alone, as suggested by incomplete adherence to the new defaults in our study. One reason for this incomplete adherence may have been inconsistent use of the tonsillectomy order set on the pediatric otolaryngology service during the postintervention period. Another reason may have been the lack of a concurrent announcement by the chief of pediatric otolaryngology that the new standard on the pediatric otolaryngology service was to prescribe 12 opioid doses. The combination of such an announcement, coupled with reinforcement of the new standard via the default settings, could have resulted in even greater reductions in opioid prescribing than observed in our study. Changing the standard alone may also be insufficient. Although the standard for the pediatric otolaryngology service was to prescribe 30 doses for adolescents and young adults undergoing tonsillectomy, the mean (SD) number of doses prescribed during the pre-intervention period was 22.3 (7.4) doses. This finding suggests that some clinicians were unaware of the standard or deliberately chose to prescribe fewer doses.

Several of our design decisions warrant further exploration. First, we did not require justification for overriding the default settings. Future studies could assess whether doing so improves adherence without impeding clinical workflow. Second, we also did not require justification for prescribing opioids in the first place, a requirement that some may feel is warranted, given that randomized clinical trials suggest that ibuprofen and opioids are equally effective for analgesia after pediatric tonsillectomy.^30^ The association between such a requirement and patient-reported outcomes could be evaluated in future research.

### Strengths and Limitations

This study has several strengths, including its inclusion of pediatric patients, strong quasi-experimental design, use of an evidence-based default dosing setting, and robust assessment of patient-reported outcomes. However, this study also has some limitations. First, owing to limited sample size, the study lacked the power to detect small changes in outcomes and did not correct for multiple comparisons. Second, conclusions regarding patient-reported outcomes may have differed had clinician adherence to the new defaults been greater. Third, survey results may have been subject to recall bias, nonresponse bias, or social desirability bias. Fourth, spillover effects to the general otolaryngology service theoretically may have occurred because residents rotate between services. However, no general otolaryngology patients in the postintervention period had 12 doses in their discharge opioid prescription.

## Conclusions

In this nonrandomized clinical trial, lowering the default number of doses in opioid prescriptions was associated with a reduction in opioid prescribing but no change in pain control. Owing to its low cost, this intervention may be easy to implement in other surgical settings. When implementing the intervention, clinicians ideally would use evidence-based settings and carefully evaluate for unintended outcomes.

## Notes

Supplement 1.
Trial Protocol

Supplement 2. **eAppendix 1.** Changes From Original Study Protocol **eAppendix 2.** Details on Opioid-Sparing Pathway **eAppendix 3.** Study Design **eAppendix 4.** Details on Intervention **eAppendix 5.** Instrument for Two-Week Survey on Postoperative Day 14 **eAppendix 6.** Details on the Analysis of Outcomes in the Secondary Analysis **eTable 1.** Characteristics of Patients in the Primary Analysis, by Treatment Group and Postintervention Status **eTable 2.** Characteristics of Patients in the Secondary Analysis **eTable 3.** Number of Patients Excluded From Each Analysis in the Secondary Analysis Owing to Missing Data **eTable 4.** Proportion of Pediatric Otolaryngology Patients in the Postintervention Period With 12 Doses in the Discharge Opioid Prescription, by Patient and Clinician Factors

Supplement 3.
Data Sharing Statement
