# Supplementary material for: Association Between Default Number of Opioid Doses in Electronic Health Record Systems and Opioid Prescribing to Adolescents and Young Adults Undergoing Tonsillectomy
Source: JAMA Netw Open. 2022 Jun 30;5(6):e2219701. doi: 10.1001/jamanetworkopen.2022.19701 (PMC9247741; doi:10.1001/jamanetworkopen.2022.19701)
Supplement: Supplement 1. — Trial Protocol [file jamanetwopen-e2219701-s001.pdf]

Default Dosing Settings for Opioid Prescriptions to Adolescents and Young Adults after Tonsillectomy

Principal Investigator:
Kao-Ping Chua, M.D., Ph.D.
Assistant Professor of Pediatrics
Susan B. Meister Child Health Evaluation and Research Center
Department of Pediatrics
University of Michigan Medical School

Funding Mechanism: Career development award to Dr. Chua from National Institute on Drug Abuse (grant number 1K08DA048110-01).

Institution(s): University of Michigan Medical School

Other Identifying Numbers: AWD012194, HUM00159821

Day Month Year: 16 September 2019

TABLE OF CONTENTS

Page

Title Page…1
Table of Contents ... 2
Signature Page ... 3
List of Abbreviations ... 4
1 Background/Scientific Rationale ... 5
2 Objectives ... 5
3 Expected Risks/Benefits ... 6
4 Eligibility ... 6
5 Subject Enrollment ... 7
6 Study Design and Procedures ... 7
7 Data Collection and Management Procedures ... 9
8 Data Analysis ... 10
9 Quality Control and Quality Assurance ... 10
10 Statistical Considerations ... 11
11 Regulatory Requirements ... 11
11.1 Informed Consent ... 11
11.2 Subject Confidentiality ... 12
11.3 Unanticipated Problems ... 13
12 References ... 16

SIGNATURE PAGE

The signature below constitutes the approval of this protocol and the attachments, and provides the necessary assurances that this study will be conducted according to all stipulations of the protocol, including all statements regarding confidentiality, and according to local legal and regulatory requirements and applicable US federal regulations.

Site Investigator:*

Signed: Kao-Ping Chua   Date: August 21, 2019

Name: Kao-Ping Chua
Title: Assistant Professor of Pediatrics, University of Michigan Medical School

** The protocol should be signed by the clinical site investigator who is responsible for the day to day study implementation at his/her specific clinical site.*

LIST OF ABBREVIATIONS

EHR	Electronic health record
NIDA	National Institute on Drug Abuse
AE	Adverse events
SAE	Serious adverse events
UM	University of Michigan

1 BACKGROUND/SCIENTIFIC RATIONALE

- Prescription opioid use by adolescents and young adults aged 12-25 years increases their risk of prescription opioid misuse.[1,2] Leftover opioids from prior prescriptions for acute pain are a key driver of this misuse. As such, it is crucial to ensure that quantities in opioid prescriptions match the needs of adolescents and young adults.[3]

- Several prior studies have utilized default dosing settings to reduce the size of opioid prescriptions written to adults. In a study in the University of Pennsylvania adult emergency department, the default quantity of oxycodone-acetaminophen tablets in the electronic health record (EHR), was set to 10 tablet; providers could override the settings and prescribe other quantities at their discretion. After this intervention was implemented, the percentage of Percocet prescriptions written for 10 tablets increased from 20.6% to 43.3%, demonstrating that EHR default settings can affect opioid prescribing.[4]

- However, no study has utilized default dosing settings to promote appropriate opioid prescribing to adolescents and young adults. Furthermore, no study of default dosing settings to date has chosen the level of these settings based on evidence on patient need.

- In this study, we will collect data on patient-reported post-surgical opioid consumption among adolescents and young adults undergoing tonsillectomy at the University of Michigan. These data will inform the design of a default dosing setting intervention. We will assess whether implementation of the intervention is associated with changes in opioid prescribing and consumption. Additionally, we will assess for potential unintended consequences, including worsened patient satisfaction, pain control, sleep, anxiety, depression, and functioning; or increased requests for refills or return visits for uncontrolled pain.

- The research team has significant experience with collecting data on patient-reported opioid consumption and patient-reported outcomes such as pain control, as well as with developing evidence-based opioid prescribing guidelines.

2 OBJECTIVES

- To evaluate changes in opioid prescribing, consumption, patient-reported outcomes, and health care utilization after implementation of a behavioral intervention using evidence-based default dosing settings for opioid prescriptions to adolescents and young adults undergoing tonsillectomy

3 EXPECTED RISKS/BENEFITS

- There are no expected direct benefits to subjects. The expected risks for subjects are no more than minimal.
  - The most likely risk to study subjects is the time burden associated with completing the surveys.
  - There is a possible risk of breach of confidentiality, although the study team will take measures to prevent such breaches.
  - There is a possible risk of worsened pain control, although literature suggests this is unlikely. For example, an evidence-based opioid prescribing guideline was instituted for University of Michigan patients undergoing gallbladder removal in 2015 and 2016. Prior to institution of this guideline, patients received an average of 50 pills of hydrocodone/acetaminophen but only consumed 6. Institution of the guideline was associated with a 63% reduction in prescription size without increasing the need for opioid prescription refills.[5]
  - There is a possible risk that the intervention could cause depression or suicidal ideation, or that increased opioid misuse could occur. Prior studies evaluating interventions to reduce opioid prescribing for acute pain have not evaluated these outcomes, so these risks remain theoretical. However, the primary mechanism of these unintended consequences would be worsened pain control, which is unlikely given the reasons above.

- Future patients and society may benefit from the research results. The default setting intervention could be broadly applicable to other procedures and conditions, and could also be readily scalable to other institutions due to its low cost of implementation. If this intervention is successful in limiting excessive opioid prescribing to adolescents and young adults, widespread adoption could reduce the number of unused opioids available for misuse and diversion in adolescents and young adults.

4 ELIGIBILITY

- Subject population: Adolescents and young adults undergoing tonsillectomy

- Inclusion criteria
  - Adolescents and young adults aged 12-25 years undergoing tonsillectomy at C.S. Mott Hospital, University Hospital, Brighton Center for Specialty Care, or Livonia Center for Specialty Care.
  - Surgeon belongs to the Department of Otolaryngology at the University of Michigan Medical School

- Exclusion criteria for primary analysis analyzing prescribing
  - Patients with prescription opioid use prior to surgery

    - Patients undergoing additional procedures at the same time as tonsillectomy (other than minor procedures such as tympanostomy tube placement)
    - Patients enrolled in another study
    - Patients who are not prescribed opioids post-operatively
    - Patients who are enrolled in the “M-POP” pathway, which includes education about pain management and a small initial opioid prescription (10 doses)
    - Patients with hospitalization with length of stay > 1 day after surgery

- Additional exclusion criteria for secondary analysis examining patient-reported outcomes through surveys
    - Patients who do not give consent (cannot reach them to enroll, decline to enroll, or non-English speaking)
    - Patients who do not complete the baseline and post-operative day 14 surveys

- Inclusion of vulnerable populations: The purpose of this study is to evaluate an intervention on opioid prescribing to adolescents and young adults. As such, minors must be included in the study.

5 SUBJECT ENROLLMENT

- Screening
    - Patients scheduled to undergo tonsillectomy during the study period will be identified using the surgical scheduling system and screened to against age criteria.

- Enrollment
    - The study team will recruit all patients meeting inclusion criteria. Recruitment will occur in-person on the day of surgery for patients undergoing tonsillectomy at C.S. Mott Hospital or University Hospital. A study team member will provide patients who enroll with a paper pain journal they can use to record the number of opioid doses consumed.
    - Recruitment will occur over the phone during the 14-day period before the day of surgery for patients undergoing tonsillectomy at the Brighton Center for Specialty Care or Livonia Center for Specialty Care. A study team member will e-mail patients who enroll with a PDF version of a pain journal they can print to record the number of opioid doses consumed.

- Incentives
    - Subjects will be compensated $10 for completing the baseline survey, $10 for completing all 13 daily post-operative surveys, and $10 for completing the final survey on post-operative day 14. Subjects will be compensated based on the number of sets of surveys completed (e.g.,

if they complete the baseline and post-operative surveys, they will receive $20). Compensation will be mailed after post-operative day 14.

6 STUDY DESIGN AND PROCEDURES

- Study design
    - The study will use a difference-in-differences design in which changes before and after the intervention will be compared between a treatment group and a control group.
    - The treatment group will include all patients undergoing tonsillectomy at C.S. Mott Hospital by a pediatric otolaryngology faculty member at the University of Michigan.
    - The control group will include all patients undergoing tonsillectomy at University Hospital, Brighton Center for Specialty Care, or Livonia Center for Specialty Care by a general otolaryngology faculty member at the University of Michigan.

- Data collection procedures throughout the study
    - There will be three sets of surveys: a baseline survey, daily surveys on post-operative days 1-13, and a final survey on post-operative day 14. Post-operative surveys will be sent to all patients who enroll, including those who are not prescribed opioids post-operatively. These patients will not be included in the primary or secondary analysis but will be analyzed in a separate study examining pain recovery without opioids.
    - Baseline survey: Patients will be asked to complete a 10-15 minute survey assessing demographics, reason for tonsillectomy, current pain, chronic pain, history of opioid use, history of opioid misuse, family chronic opioid use, depression, substance use disorders, anxiety, sleep, and global health. For patients at Mott or University Hospital, the baseline survey will be administered by a study team member in-person through Qualtrics on a tablet. Patients at Brighton or Livonia will be e-mailed a link to the baseline survey upon enrollment; for patients who cannot receive e-mail links, the survey will be administered over the phone.
    - Daily surveys: At 12:01 am on post-operative days 1 through 13, patients will be e-mailed a daily link to complete a 1-minute online Qualtrics survey in which pain and opioid consumption over the prior 24 hours will be assessed. The post-operative day 7 survey will be similar except that it will include additional questions assessing pain over the prior 7 days. Patients who cannot receive e-mail links will not be sent daily surveys.
    - Final survey: on post-operative day 14, patients will be e-mailed a link to complete a 10-15-minute online Qualtrics survey assessing pain control, opioid use, opioid disposal, opioid misuse, acetaminophen/ibuprofen use, depression, anxiety, sleep, and health care utilization during the 14 days after surgery, including refills and

visits to the emergency department or primary care for pain. Patients who cannot receive e-mail links will be called and the survey will be administered over the phone.
  - DataDirect, the University of Michigan's clinical data warehouse, will be queried to determine patient demographics, the dosing parameters of post-operative opioid prescriptions, the number of return visits for pain at the University of Michigan in the 14 days after surgery, and the number of opioid refills in the 14 days after surgery. The baseline survey will also assess demographics but not all patients will enroll and complete this survey; therefore, DataDirect analysis will be necessary.
  - MiChart, the University of Michigan's EHR implementation of Epic, will be used to determine opioid use prior to the day of surgery based on the admission history and physical. The baseline survey will also assess recent opioid use but not all patients will enroll and complete this survey; therefore, MiChart analysis will be necessary.

- Timeline
  - The study will occur over the course of a 13-month period. The pre-intervention period will be the seven-month period between October 1, 2019 to April 30, 2020, while the post-intervention period will be the six-month period between May 1, 2020 and October 31, 2020.
  - The intervention will be implemented on May 1, 2020.

- Nature of intervention
  - Currently, pediatric otolaryngology attendings and their residents use a MiChart tonsillectomy orderset with three options: patients < 12 years, patients ≥12 years, and M-POP. Under the orderset for patients ≥12 years, the default is currently 30 doses, consistent with the current departmental prescribing standard (see MiChart screenshot below).
  - During the seven-month pre-intervention period, no changes will be made to the default number of doses or any other dosing parameter.
  - During the month of April 2020 (month 7), opioid consumption data will be reviewed by the chief of pediatric otolaryngology. A new default number of doses lower than 30 will be chosen based on the level and distribution of opioid consumption.
  - On May 1, 2020, the default number of doses in the tonsillectomy orderset will be changed to the new default <u>at Mott only.</u> Thus, all patients in the treatment group (patients undergoing tonsillectomy at Mott by pediatric otolaryngology attendings) will have clinicians who are exposed to the new default settings.
  - The tonsillectomy orderset will <u>not</u> be changed on May 1, 2020 at University Hospital, Brighton, or Livonia. Thus, all patients in the control group (patients undergoing tonsillectomy in these locations by general otolaryngology attendings) will have clinicians who will not be exposed to the new default settings, even if they use the pediatric otolaryngology tonsillectomy ordeset.

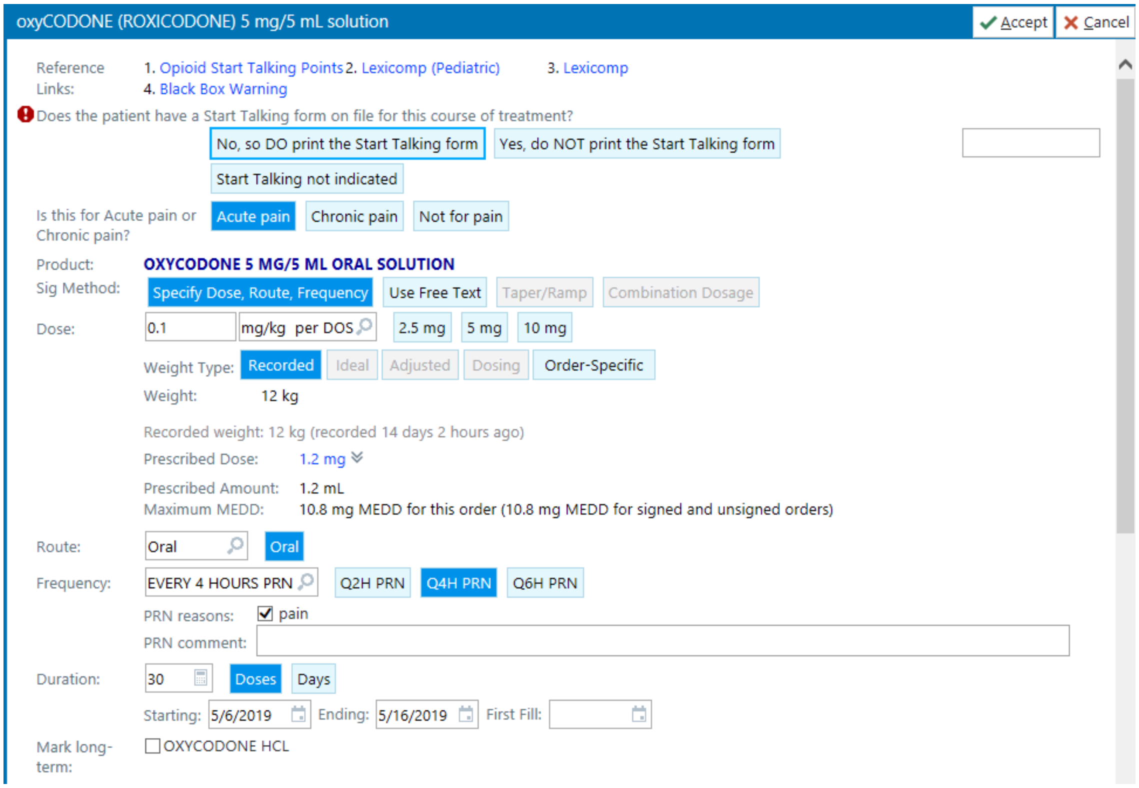

7 DATA COLLECTION AND MANAGEMENT PROCEDURES

- As noted above, the baseline survey will be administered via Qualtrics on a tablet in-person for patients at Mott or University Hospital, or via an online Qualtrics survey for patients at Brighton or Livonia. Daily surveys on post-operative days 1-13 will be administered via an online Qualtrics survey, as will the final post-operative day 14 survey (with the exception of patients who cannot receive e-mail links, who will be administered the final survey over the phone).

- Telephone follow-up will occur for patients who do not complete the post-operative day 14 survey within 3 days. Telephone follow-up will not occur for the daily surveys on post-operative days 1-13.

- Data entry
    - Survey data obtained through Qualtrics will automatically be uploaded into a secure database.
    - All data extracted from DataDirect and MiChart will be manually entered into this secure database.

- Data management and security

- Only study team members trained in human subjects protection will have access to study data
- Only coded patient identifiers will be associated with study data. Only the PI will have the crosswalk between these identifiers and identifiable information such as patient name.
- Electronic data files will be password-protected and stored on a secure server with restricted access.
- Data will be destroyed within 90 days of completion of the study.

8 DATA ANALYSIS

- Data analysis will occur at the end of the 13-month study period.

- The primary analysis will examine changes in opioid prescribing and health care utilization among all patients who meet the inclusion and exclusion criteria listed in section 4.

- The secondary analysis will additionally examine changes in patient-reported outcomes among patients who consent to enroll in the study and meet the exclusion criteria listed in section 4.

9 QUALITY CONTROL AND QUALITY ASSURANCE

- Data on patient-reported outcomes will be collected using standardized measures that have been validated in other samples.

- On a monthly basis, the project coordinator will print frequency data from surveys and examine for unexpected results or patterns of missingness. Any issues will be resolved with the PI.

- The PI and the project coordinator will ensure all survey data are backed-up on UM’s secure network, and will also ensure that data are password-protected and only accessible to the research team.

- The project coordinator will ensure that all participants are accounted for in tracking database.

- Each month, participant IDs will randomly be selected to ensure that consents have been obtained.

10 STATISTICAL CONSIDERATIONS

- Outcomes in primary analysis
  - Number of doses in the discharge opioid prescription

- Proportion of patients in which the number of doses in the discharge opioid prescription equals the number of doses in the new default settings
- Proportion of patients prescribed opioids at discharge
- Proportion of patients prescribed opioids at discharge who had at least one opioid prescription refill from UM during the two weeks after surgery
- Proportion of patients who saw a UM provider in an office due to pain during the two weeks after surgery
- Proportion of patients with at least one emergency department visit or hospitalization at UM for pain during the two weeks after surgery

- Additional outcomes in secondary analysis (based on data from the final post-operative day 14 survey)
  - Overall satisfaction with pain control during the two weeks after surgery (scale of 1-10)
  - Proportion of patients who reported that their pain was well-controlled
  - Proportion of patients whose overall pain control was rated as much worse or worse than expected
  - Proportion of patients whose pain has resolved by day 14 after surgery
  - Pain score over the past 7 days at its worst (scale of 0-10)
  - Pain score over the past 7 days on average (scale of 0-10)
  - Number of opioid doses taken during the two weeks after surgery
  - Proportion of patients with leftover doses of opioids
  - Proportion of patients with leftover doses who disposed of them
  - Proportion of patients who misused opioids belonging to others at least once during the two weeks after surgery
  - Proportion of patients who misused their own opioids at least once during the two weeks after surgery
  - PHQ-8 score (depression)
  - Score on PROMIS Pediatric Anxiety – Short Form 8a
  - Score on PROMIS Sleep Disturbance – Short Form 4a
  - Proportion of patients who saw their primary care provider (or a colleague) in the office due to poorly controlled pain at least once during the two weeks after surgery
  - Proportion of patients who visited an urgent care center due to poorly controlled pain at least once during the two weeks after surgery
  - Proportion of patients who visited a retail clinic due to poorly controlled pain at least once during the two weeks after surgery
  - Proportion of patients who went to an emergency department and/or were hospitalized due to poorly controlled pain at least once during the two weeks after surgery

- Data from the daily surveys will be used to assess whether daily opioid consumption reports matches the consumption reported on the final post-

operative day 14, and will also be analyzed in a separate study assessing the trajectory of pain and opioid use after tonsillectomy.

- We will fit linear or logistic regression models assessing outcomes as a function of an indicator of post-intervention status, an indicator of treatment group status, and its interaction, controlling for covariates such as demographics (e.g., age, sex) and baseline co-morbidities (e.g., depression, anxiety). We will graphically and statistically assess the comparability of pre-intervention monthly trends in outcomes between the treatment and control groups.

- We hypothesize that there will be no change in outcomes in the control group (i.e., that the post-intervention trend in outcomes in the control group will be an extension of the pre-intervention trend in outcomes). In contrast, we hypothesize that the number of doses and opioid consumption will drop in the treatment group after the intervention; that the proportion of patients with prescriptions concordant with the new defaults will increase after the intervention; and that there will be no change in balancing measures such as pain control or depression.

- Sensitivity analyses
    - Statistical modeling: we will explore whether results change when replacing the indicator of treatment group status with a vector of fixed effects for the prescribing clinician, and will explore the feasibility of using an interrupted time series approach instead of a difference-in-differences approach.
    - We will determine whether any otolaryngology residents wrote opioid prescriptions for both a pediatric otolaryngology and general otolaryngology patients in the post-intervention period, and exclude data from any such residents from the analysis
    - We will exclude opioid prescriptions written for patients of the chief of pediatric otolaryngology, who will be involved in setting the new defaults

- Subgroup analysis will occur by age group (ages 12-17 years vs 18 and older), gender, payer type (private/public), and baseline co-morbidities (e.g., depression and anxiety).

- We will separately analyze data from pediatric otolaryngology patients undergoing surgery outside of Mott and adult otolaryngology patients undergoing surgery at Mott to assess for any spillover effects.

- Sample size: Based on historical UM data, we estimate that there will be 113 patients in the treatment group and 85 in the control group for the primary analysis (total 198). If 65% consent to complete the surveys, then there will

be 74 and 55 patients in the treatment and control groups for the secondary analysis, respectively (total 129).

11 REGULATORY REQUIREMENTS

11.1 Informed Consent

- For patients recruited in-person, a study team member will obtain written consent from young adults aged ≥18 years. For minors, written consent will be obtained from a parent and verbal assent will be obtained from the minor.

- For patients recruited over the phone, a study team member will obtain verbal consent from young adults. For minors, the study team member will obtain verbal consent from a parent and verbal assent from the minor.

- Consent will be obtained via a document approved by IRBMED, the University of Michigan Medical School Institutional Review Board. The document will include an explanation of the study, including the purpose, testing procedures, time commitment, inclusion/exclusion criteria, risks and benefits, confidentiality, compensation, study personnel contacts, and required regulatory information. All individuals will be given the opportunity to ask questions and participant understanding will be assessed.

- Minors will be assured that their responses will not be shared with anyone else except in limited circumstances (e.g., imminent self-harm).

11.2 Subject Confidentiality

- Measures to ensure confidentiality
    - Following informed consent, the study participant will be assigned a coded study identification number. From this point forward, the coded participant identification number will be the primary means of identifying the participant.
    - All study staff will be trained, and physical security measures for data capture and storage will be used. A minimum of identifiable information will be collected. Records will be kept confidential to the extent provided by Federal, State, and local law.
    - Subjects will be informed that the sponsor and the Institutional Review Board may inspect the records of this investigation.
    - All participants will have direct access to the phone numbers and pagers of the study coordinator and responsible investigators, as well as a 24-hour contact number.
    - Participants will not be identified in any reports on this study.

- Justification for use of personally identifiable data or private health information (PHI)
    - Personally identifiable data (e.g., patient name) is necessary to extract information from DataDirect and MiChart.

- Certificate of Confidentiality
    - Because this is an NIH-funded study, a Certificate of Confidentiality will automatically be issued.

- Exceptions to Confidentiality
    - In the consent document, the following will statement be included: “If we learn that you have severe depression or might be misusing your pain medications or others’ pain medications, a mental health professional on our team will contact you. If you are a minor and we learn that you are at immediate risk of harming yourself, we may contact your parent or guardian.”
    - Furthermore, it will be stated that “The researchers will use the Certificate to resist any demands for information that would identify you, except if we learn of information about suspected or known sexual, physical, or other abuse of a child or older person, or threats of violence to self or others. If any member of the research team is given such information, he or she will make a report to the appropriate authorities; or if personnel of the United States federal or state government agency sponsoring the project requests information for auditing or program evaluation to meet the requirements of the federal Food and Drug Administration (FDA).

11.3 Unanticipated Problems

- According to IRBMED, reportable adverse events (AEs) are “any experience or abnormal finding that has taken place during the course of a research project and was harmful to the subject participating in the research, or increased the risks of harm in the research, or had an unfavorable impact on the risk/benefit ratio.” In this context of this study, AEs include violation of confidentiality and significantly worsened pain control. Serious adverse events (SAEs) include suicidal ideation or opioid misuse resulting in overdose.

- Procedures for Reporting Adverse Events
    - *IRBMED.* AEs and SAEs related to study intervention will be reported to IRBMED. The timing of the reporting will be dependent on the severity of the event, and whether such adverse events were expected (i.e., included in the informed consent). The study team will adhere to the IRBMED Reporting Plan regarding the timing of report. For SAEs, the initial SAE report will be submitted within 24 hours. The completed SAE report will be submitted in writing within

7 days. Non-life threatening AEs that are causally related to the research (e.g., breach in confidentiality) will be reported in writing within 14 days. For incidents at the level of AE or SAEs, Dr. Chua will be notified by study staff as soon possible, and Dr. Chua will be responsible for reporting the event to IRBMED on the timeline described above.

    - *NIDA.* All significant actions by IRBMED (including any correspondence related to SAEs) will be communicated directly to the NIDA program officer within 24 hours of learning of their occurrence. Additionally, all yearly reports to NIDA will include a summary of all relevant correspondences with IRBMED during the past year. AEs will be reported to the NIDA program officer at least once per year during an annual progress report. This report will describe the AE event, when it occurred, and the outcome/resolution. If there were no AEs, the study team will submit a statement to the program officer in writing that no AEs occurred. In the event of an SAE, an initial report including a brief explanation of the event and when it occurred will be given to the NIDA program officer via email within 24 hours of learning of the event. Within 72 hours, the study team will provide a written follow-up including the following details: date of the event, what occurred, actions taken by the project staff, planned follow up, whether the event appears related to the intervention, and whether the participant will continue in the study.

- Managing Participant Distress
    - Participants will be made aware of their right to refuse to answer any questions that make them uncomfortable or that they do not wish to answer, and they will be informed of their right to withdraw from the study at any time without penalty. This message will be conveyed during the informed consent process. All participants will have direct access to the phone number and e-mails of the study coordinator and PI.

- Managing Participant Safety
    - We will monitor pain control after surgery through the post-operative surveys described in the proposal. Of note, most post-tonsillectomy pain resolves within two weeks. We plan to conduct the post-operative surveys two weeks after surgery, so we do not anticipate that patients will still be in pain at the time the surveys are administered. However, if patients do report poor pain control, we will contact the patient’s surgical team, who will follow up with the patient and prescribe opioids or bring the patient in for evaluation, as appropriate. Electronic prescribing is currently available at the University of Michigan, so patients will not necessarily have to come back in for a visit to obtain refills. In short,

we do not anticipate that our intervention will worsen pain control, and have a specific plan for addressing this contingency were it to occur.
    - In the baseline and post-operative day 14 surveys, we will administer the PHQ-8, a depression screen that does not contain any questions regarding self-harm. These surveys will predominantly be administered via a tablet or online Qualtrics survey. A small number of patients may be administered the surveys on the phone (e.g., if they cannot receive e-mail links to online surveys). If, in the course of these phone surveys, a patient discloses thoughts of self-harm to the study coordinator, the study coordinator will contact a mental health professional on the team, who will contact patients and screen for suicide risk using a suicidal ideation protocol developed by the University of Michigan Back and Pain Center. Patients without an imminent threat of self-harm will be referred to mental health services. For patients with an imminent threat of self-harm, we will follow the protocol by trying to keep them on the phone while paging an emergency University of Michigan-based pager. Details of the protocol are included in the data and safety monitoring plan. If the patient with imminent threat of self-harm is a minor, we will additionally inform their parents or guardians.

- Trial Stopping Rules
    - The trial will be stopped if it is discovered that the intervention has resulted in substantially higher rates of uncontrolled pain, depression, or opioid misuse. This is felt to be unlikely for the reasons articulated in Section 3.

12 REFERENCES

1. Miech R, Johnston L, O'Malley PM, Keyes KM, Heard K. Prescription Opioids in Adolescence and Future Opioid Misuse. *Pediatrics.* 2015;136(5):e1169-1177.
2. McCabe SE, West BT, Boyd CJ. Medical use, medical misuse, and nonmedical use of prescription opioids: results from a longitudinal study. *Pain.* 2013;154(5):708-713.
3. McCabe SE, West BT, Boyd CJ. Leftover prescription opioids and nonmedical use among high school seniors: a multi-cohort national study. *J Adolesc Health.* 2013;52(4):480-485.
4. Delgado MK, Shofer FS, Patel MS, et al. Association between Electronic Medical Record Implementation of Default Opioid Prescription Quantities and Prescribing Behavior in Two Emergency Departments. *J Gen Intern Med.* 2018.
5. Howard R, Waljee J, Brummett C, Englesbe M, Lee J. Reduction in Opioid Prescribing Through Evidence-Based Prescribing Guidelines. *JAMA Surg.* 2018;3(153):286-287.
