# Supplementary material for: Association Between Default Number of Opioid Doses in Electronic Health Record Systems and Opioid Prescribing to Adolescents and Young Adults Undergoing Tonsillectomy
Source: JAMA Netw Open. 2022 Jun 30;5(6):e2219701. doi: 10.1001/jamanetworkopen.2022.19701 (PMC9247741; doi:10.1001/jamanetworkopen.2022.19701)
Supplement: Supplement 2. — eAppendix 1. Changes From Original Study Protocol eAppendix 2. Details on Opioid-Sparing Pathway eAppendix 3. Study Design eAppendix 4. Details on Intervention eAppendix 5. Instrument for Two-Week Survey on Postoperative Day 14 eAppendix 6. Details on the Analysis of Outcomes in the Secondary Analysis eTable 1. Characteristics of Patients in the Primary Analysis, by Treatment Group and Postintervention Status eTable 2. Characteristics of Patients in the Secondary Analysis eTable 3. Number of Patients Excluded From Each Analysis in the Secondary Analysis Owing to Missing Data eTable 4. Proportion of Pediatric Otolaryngology Patients in the Postintervention Period With 12 Doses in the Discharge Opioid Prescription, by Patient and Clinician Factors [file jamanetwopen-e2219701-s002.pdf]

Supplementary Online Content

Chua KP, Thorne MC, Ng S, Donahue M, Brummett CM. Association between default number of opioid doses in electronic health record systems and opioid prescribing to adolescents and young adults undergoing tonsillectomy. *JAMA Netw Open.* 2022;5(6):e2219701. doi:10.1001/jamanetworkopen.2022.19701

eAppendix 1. Changes From Original Study Protocol
eAppendix 2. Details on Opioid-Sparing Pathway
eAppendix 3. Study Design
eAppendix 4. Details on Intervention
eAppendix 5. Instrument for Two-Week Survey on Postoperative Day 14
eAppendix 6. Details on the Analysis of Outcomes in the Secondary Analysis
eTable 1. Characteristics of Patients in the Primary Analysis, by Treatment Group and Postintervention Status
eTable 2. Characteristics of Patients in the Secondary Analysis
eTable 3. Number of Patients Excluded From Each Analysis in the Secondary Analysis Owing to Missing Data
eTable 4. Proportion of Pediatric Otolaryngology Patients in the Postintervention Period With 12 Doses in the Discharge Opioid Prescription, by Patient and Clinician Factors

This supplementary material has been provided by the authors to give readers additional information about their work.

© 2022 Chua KP et al. *JAMA Network Open.*

eAppendix 1. Changes From Original Study Protocol

Our initial plan was to implement the intervention in May 2020 and recruit patients during a 13-month study period from October 2019 to October 2020. However, owing to the COVID-19 pandemic, elective procedures were suspended at the University of Michigan from late March 2020 to late May 2020. Even after procedures resumed, tonsillectomy volume was substantially lower compared to the pre-pandemic baseline, especially on the general otolaryngology service. As a result, we made the following changes:

- We excluded the months of April-May 2020 from analyses.
- We implemented the intervention on October 1, 2020 and extended the trial through July 31, 2021 to increase sample size.
- We obtained permission from our Institutional Review Board to include patients in the primary analysis who could not be reached for recruitment. Only the electronic health record data of these patients were examined.
- We altered our recruitment strategy so that patients could be recruited over the phone before surgery instead of in-person (as in-person contact was restricted).
- To maximize sample size for the secondary analysis, we obtained permission to follow-up with patients who had not completed the baseline and post-operative day 14 survey.

In addition to the above changes, we removed one outcome from the primary analysis that was erroneously included in the original study protocol (proportion of patients with discharge opioid prescriptions - all patients included in analyses had such prescriptions). Owing to unforeseen circumstances that arose during the course of the study, we also added the following exclusions to the study eligibility criteria: patients living in foster care or with a legal guardian, patients with medical complexity or developmental delays, patients with recent suicidal ideation documented in their chart, and patients undergoing emergent tonsillectomy.

© 2022 Chua KP et al. *JAMA Network Open.*

eAppendix 2. Details on Opioid-Sparing Pathway

At the time the study protocol was developed, the pediatric otolaryngology service had an opioid-sparing pathway called the Michigan Pain-Control Optimization Pathway (MPOP). This pathway was for patients who wished to have no opioids after tonsillectomy or a small opioid prescription. Patients who opted for this pathway were booked as an MPOP tonsillectomy in the operating room schedule. In our study, there were 2 patients booked at an MPOP tonsillectomy. Per protocol, we excluded these 2 patients from the analysis.

© 2022 Chua KP et al. *JAMA Network Open.*

eAppendix 3. Study Design

On October 1, 2020, the default number of doses in discharge opioid prescriptions was lowered from 30 to 12 in the pediatric tonsillectomy order set. The general otolaryngology service does not use the pediatric tonsillectomy order set and therefore was not exposed to the change in default settings. The change in the order set only occurred at C.S. Mott Children's Hospital, the location in which all pediatric otolaryngology patients included in the study underwent tonsillectomy. If a clinician attempted to use the order set in a different setting, the previous version of the order set displayed with the old default of 30 doses.

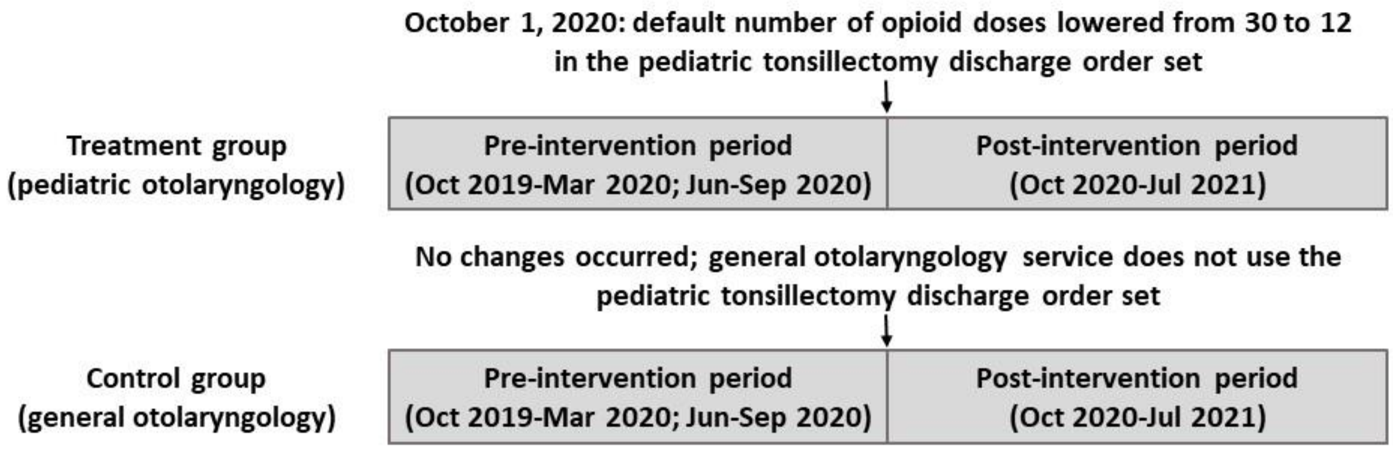

© 2022 Chua KP et al. *JAMA Network Open.*

eAppendix 4. Details on Intervention

Screenshot of the tonsillectomy order set prior to the intervention

For all patients aged 12 years and older, there was a single pathway. Within this pathway, this is the screen that would display for the opioid prescription. The default opioid prescription was oxycodone solution 0.1 mg/kg per dose by mouth every 4 hours as needed, with a default number of 30 doses. Though not shown in the image below, the default dispense quantity was not auto-populated. According to the University of Michigan's information technology team, auto-population of the dispensing quantity was not technically feasible in Epic when weight-based dosing is used. Although the image is cut off at the bottom, the default quantity was not auto-populated.

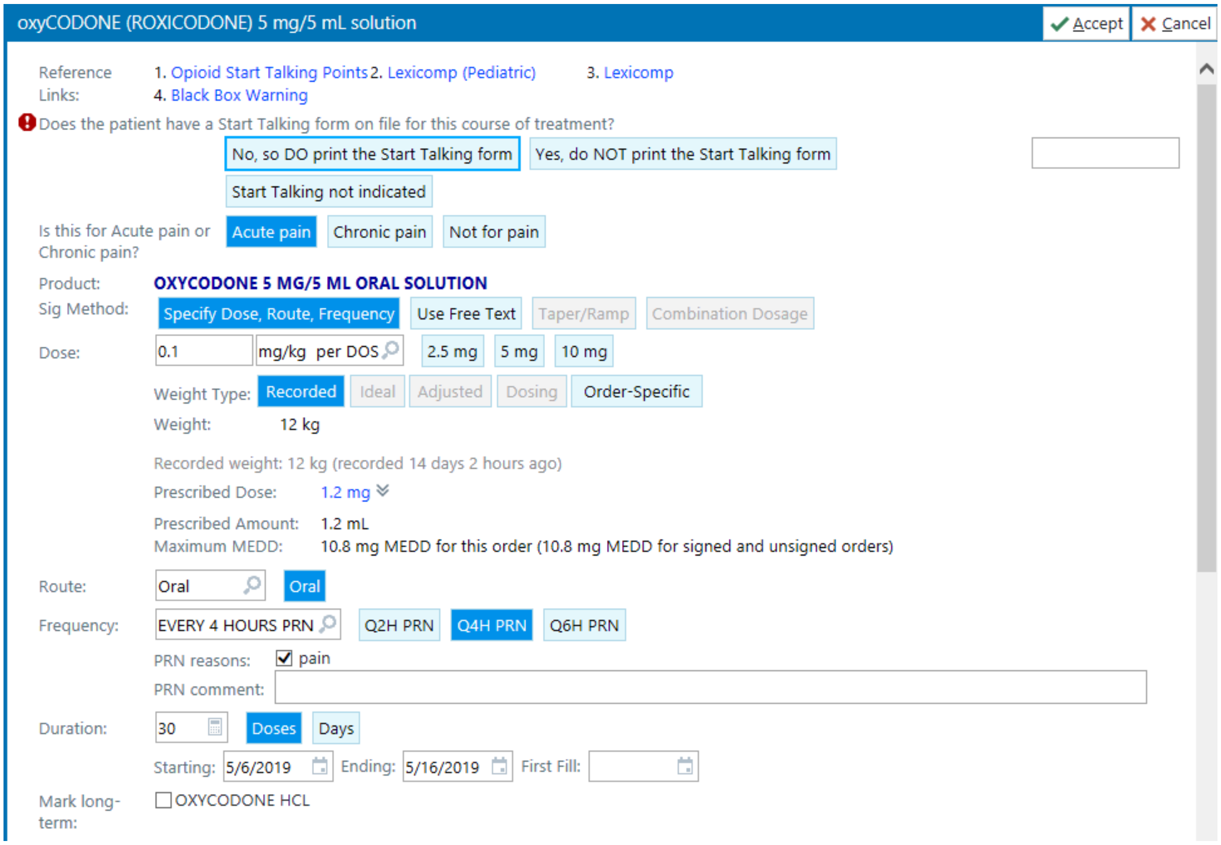

© 2022 Chua KP et al. *JAMA Network Open.*

Screenshot of the tonsillectomy order set after the intervention: option 1

After the intervention, there were three pathways for patients aged 12 years and older that could be selected. Option 2 was used when clinicians wished to prescribe oxycodone solution to patients weighing less than 50 kg, a threshold below which weight-based dosing is used. The default opioid prescription was oxycodone solution 0.1 mg/kg per dose by mouth every 4 hours as needed. The default number of doses was 12 (rather than 30). Owing to the use of weight-based dosing, it was not technically possible in Epic to auto-populate the quantity.

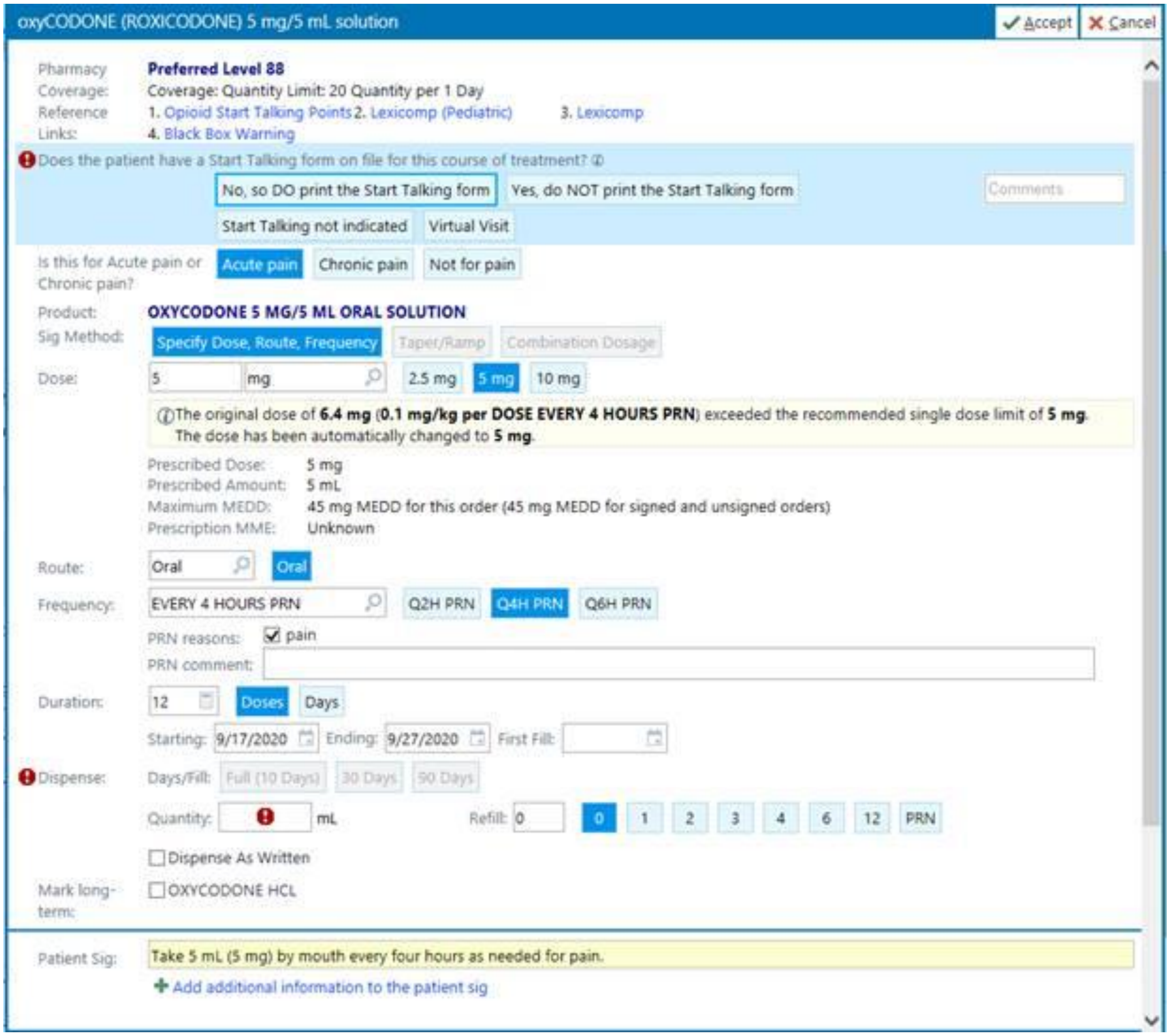

© 2022 Chua KP et al. *JAMA Network Open.*

Screenshot of the tonsillectomy order set after the intervention: option 2

Option 2 was used when clinicians wished to prescribe oxycodone solution to patients weighing ≥ 50 kg. The default opioid prescription was oxycodone solution 5 mg per dose by mouth every 4 hours as needed. The default number of doses was 12, and the default quantity was auto-populated to 60 mL.

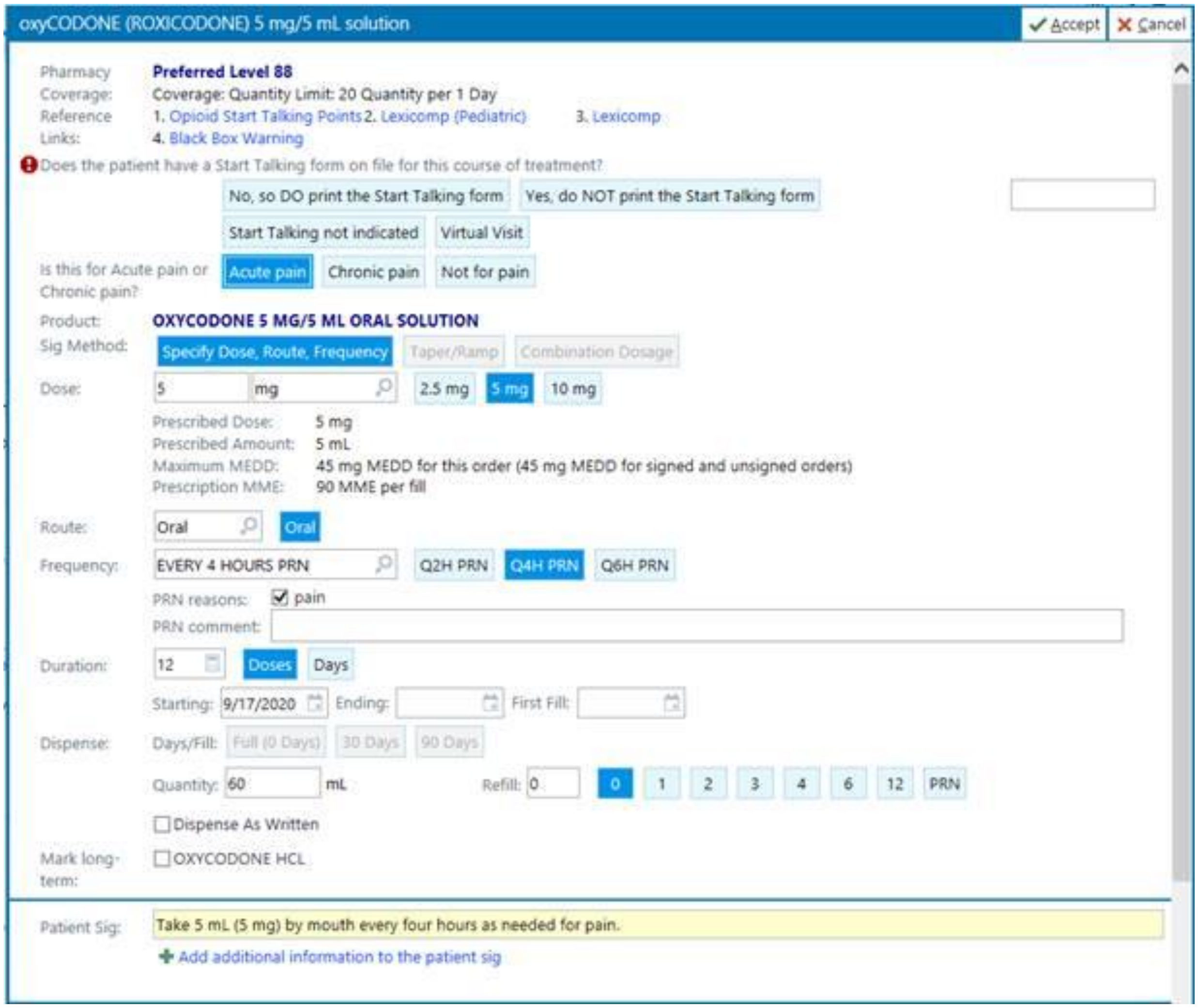

© 2022 Chua KP et al. *JAMA Network Open.*

Screenshot of the tonsillectomy order set after the intervention: option 3

Option 3 was used when clinicians wished to prescribe oxycodone tablets to patients weighing ≥ 50 kg. The default opioid prescription was oxycodone tablets 5 mg per dose by mouth every 4 hours as needed. The default number of doses was 12, and the default quantity was auto-populated to 12 tablets.

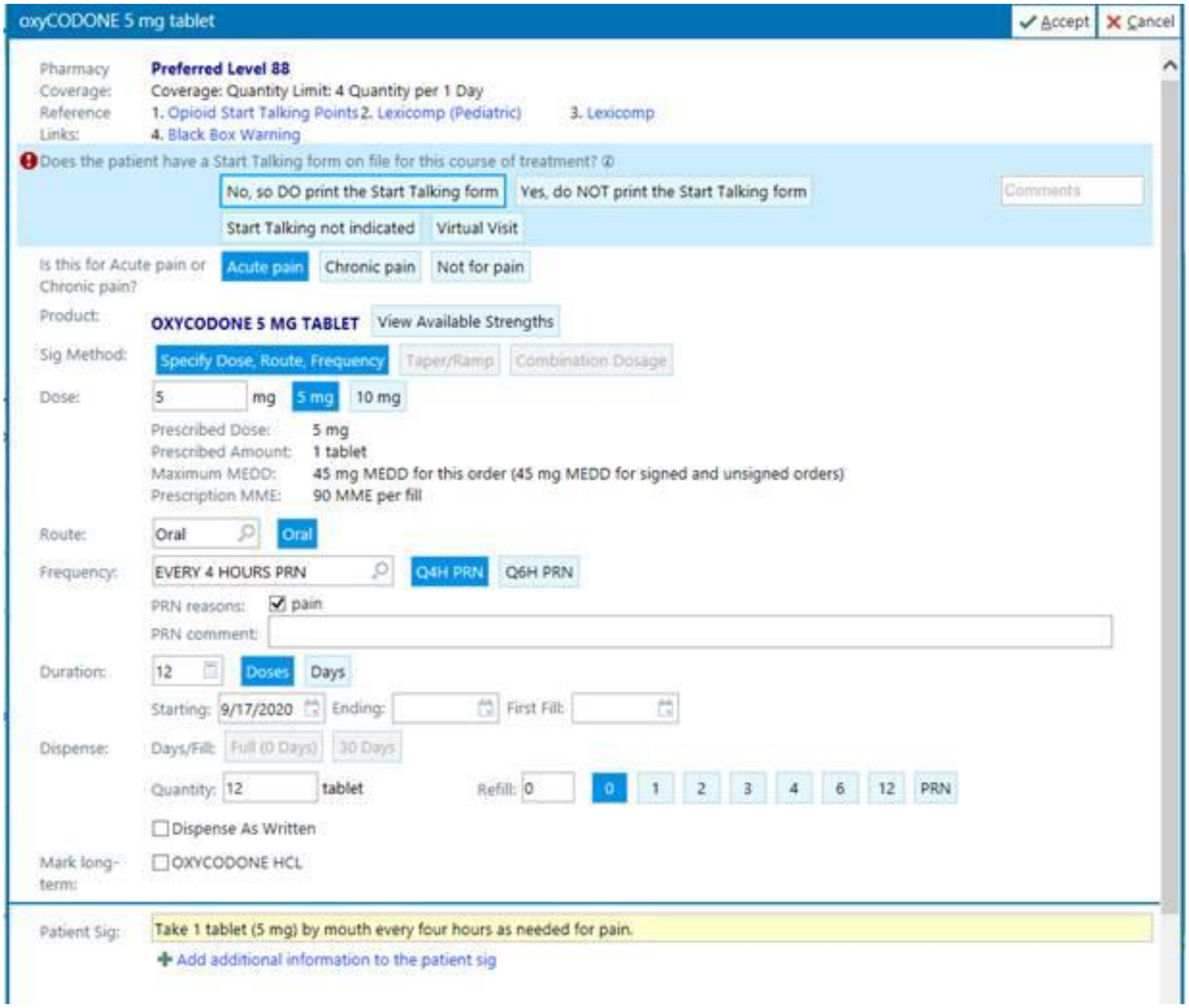

© 2022 Chua KP et al. *JAMA Network Open.*

eAppendix 5. Instrument for Two-Week Survey on Postoperative Day 14

This survey will ask you about your pain control, pain medication use, experiences, and health care visits after your tonsils were removed. Some of these questions will be asking you about things that are usually kept private. Remember, this survey is completely confidential. This means that your name will not be connected with your answers. Your parents/guardians, friends, or anyone else you know will **never** be able to see your responses. You can choose to not answer questions or quit the survey at any time. By clicking “Next”, you are showing us that you understand that your answers are confidential, and that you can stop the survey at any time without anyone knowing that you quit the survey.

© 2022 Chua KP et al. *JAMA Network Open.*

These six questions refer to pain in your mouth or throat during the two weeks after surgery.

1. Overall, how would you rate your satisfaction with how well your pain was controlled during the two weeks after surgery?

1	2	3	4	5	6	7	8	9	10
Very dissatisfied									Very satisfied

2. During the two weeks after surgery, would you say your pain has been poorly controlled, adequately controlled, or well-controlled?

Poorly controlled
Adequately controlled
Well-controlled
Don't know/prefer not to say

3. How would you describe how well your pain was controlled during the two weeks after surgery?

Much worse than you expected
Worse than you expected
About what you expected
Better than you expected
Much better than you expected
Don't know/prefer not to say

4. How many days after surgery did your pain stop?

0 (pain stopped on the day of surgery)
1
2
3
4
5
6
7
8
9
10
11
12
13
14 days or more
I still have pain
Don't know/prefer not to say

5. Please describe the pain in your mouth or throat over the past 7 days **at its worst**. Use the scale below, where 0 indicates “no pain” and 10 indicates the “worst pain you could possibly imagine”.

© 2022 Chua KP et al. *JAMA Network Open.*

0 1 2 3 4 5 6 7 8 9 10
No pain

6. Please describe the pain in your mouth or throat over the past 7 days **on average**. Use the scale below, where 0 indicates "no pain" and 10 indicates the "worst pain you could possibly imagine".

0 1 2 3 4 5 6 7 8 9 10
No pain

These questions refer to prescriptions for opioid pain medications you might have received during the two weeks after surgery, including prescriptions before going home from surgery, prescription refills from a UM surgeon (or someone who works with a UM surgeon like a nurse) when the initial prescription after surgery ran out, and prescriptions from someone else (like a primary care or emergency department doctor). Examples of opioid pain medications include: **oxycodone** (Oxycontin, Percocet, Percodan, Roxicodone); **hydrocodone** (Vicodin, Lortab, Norco, Zohydro ER); **tramadol** (Ultram, Ultram ER, Ultracet); **codeine** (Tylenol #3, Tylenol #4); **morphine** (Avinza, Kadian, MS Contin); **fentanyl** (Duragesic, Fentora); **buprenorphine** (Suboxone); **oxymorphone** (Opana, Opana ER); **hydromorphone** (Demerol; Dilaudid); and **methadone.** Please do **not** include "over-the-counter" pain relievers that can be bought in drug or grocery stores without a prescription, such as aspirin, acetaminophen (Tylenol), ibuprofen (Advil, Motrin) or naproxen (Aleve).

7. Did you receive any prescriptions for opioid pain medications during the two weeks after surgery?

Yes
No [skip to question 17]
Don't know/prefer not to say [skip to question 17]

8. Check all that apply:

□ I received a prescription for opioid pain medications before going home from surgery [if checked, go to question 9; if not, skip question 9]
□ During the two weeks after surgery, I received a refill for opioid pain medications from a UM surgeon (or someone who works with a UM surgeon like a nurse) after the initial prescription after surgery ran out
□ During the two weeks after surgery, I received a prescription for opioid pain medications from my primary care doctor or one of his or her colleagues
□ During the two weeks after surgery, I received a prescription for opioid pain medications from a doctor or other health care professional at an emergency department
□ During the two weeks after surgery, I received a prescription for opioid pain medications from some other source (please list the source(s) here: _____)
□ Don't know/prefer not to say

9. If you received a prescription for opioid pain medications before going home from surgery, did you or a family member fill this prescription at a pharmacy?

Yes
No
Don't know/prefer not to say

10. This question refers to opioid pain medications you might have taken during the two weeks after surgery. Please include opioid pain medications from any prescription you received during the two weeks after surgery,

© 2022 Chua KP et al. *JAMA Network Open.*

Refer to the box “total number of doses during the two weeks after surgery” in your pain journal to answer this question.

How many total number of doses of opioid pain medications did you take during the two weeks after surgery? For example, if you were prescribed 5 mL of oxycodone per dose, took 5 mL of oxycodone at 2 pm on day 1 and 5 mL of oxycodone at 8 pm on day 2, and did not take any other doses after that, you took a total of two doses during the two weeks after surgery. You can use decimal points if needed (for example, 1.5 doses).

Zero doses [skip to question 12]
At least one dose (specify the number of doses: _____) [skip to question 12]
I am not sure

11. Please provide your best estimate of the number of doses of opioid pain medications you took during the two weeks after surgery.

1-5
6-10
11-15
16-20
21-25
26-30
31-35
36-40
41-45
46-50
51-55
56-60
61 or more
Don’t know/prefer not to say

12. Did you take all of the opioid pain medications that were prescribed to you during the two weeks after surgery?

Yes [if yes, skip to question 17]
No
Don’t know/prefer not to say

13. Did you (or a family member) dispose of **all** of the leftover doses of opioid pain medications (that is, all of the unused pills or all of the unused liquid opioid pain medication)?

Yes [if yes, go to question 14]
No [If no, then skip to question 15]
Don’t know/prefer not to say [skip to question 17]

14. How did you (or a family member) dispose of the leftover doses of opioid pain medications? Check all that apply.

□ Flushed down the toilet/poured down the sink
□ Thrown away as is

© 2022 Chua KP et al. *JAMA Network Open.*

□ Thrown away mixed with kitty litter or coffee grounds
□ Thrown away in a drug deactivation bag/container
□ Took the medication to an opioid take-back event
□ Took the medication to a law-enforcement agency
□ Took the medication to an authorized pharmacy or drop box
□ Other (please explain: ______)
□ Don't know/prefer not to say

[After question 14, skip to question 17]

15. Where are the leftover doses of opioid pain medications stored? Check all that apply.
□ Locked medicine cabinet
□ Unlocked medicine cabinet
□ Purse or other bag
□ Drawer (any drawer in the home)
□ Closet (any closet in the home)
□ Out in the open or on a counter
□ Other (please explain: _____)
□ Don't know/prefer not to say [skip to question 17 if checked]

16. Why were the leftover doses of opioid pain medications not disposed of? Check all that apply.
□ Do not know how to dispose of them properly
□ Plan to dispose of them and know how to do so, but have not gotten around to it yet
□ Chose to keep them in case I, my friends, or my relatives have pain in the future
□ Gave them away or loaned them to someone else
□ Sold them to someone else
□ Traded them for something else (like other medications, other drugs, clothes, etc.)
□ Someone took the leftover doses without asking me
□ Other (please explain: _____)
□ Don't know/prefer not to say

The next questions ask about using opioid pain medications in any way a doctor did not direct you to use them during the two weeks after surgery, including:

Using them without a prescription of your own (using medications that were not prescribed to you)
Using them in greater amounts, more often, or longer than you were told to take it
Using them in any other way a doctor did not direct you to use it

17. During the two weeks after surgery, did you ever, even once**,** use opioid pain medications in any way a doctor did not direct you to use them?

Yes
No [if no, then skip to question 20]
Don't know/prefer not to say [skip to question 20]

18. In what ways have you used opioid pain medications in any way a doctor did not direct you to use them during the two weeks after surgery? Check all that apply.

© 2022 Chua KP et al. *JAMA Network Open.*

□ At least once, I took opioid pain medications that were not prescribed to me [if checked, go to question 19; if not, skip to question 20]
□ At least once, I took a higher amount of opioid pain medication than a doctor directed me to (for example, taking twice as much medication per dose than my doctor directed me to)
□ At least once, I took opioid pain medications more often than a doctor directed me to (for example, taking the medications every two hours when my doctor directed me to take them every four hours)
□ I took opioid pain medication for longer (more days) than a doctor directed me to
□ I took opioid pain medication in some other way than a doctor directed me to (please explain: ______)
□ Don't know/prefer not to say

[After question 18, skip to question 20 unless first option is checked]

19. Think about the **last time** you took opioid pain medications that were not prescribed to you during the two weeks after surgery. How did you obtain these medications?
I obtained them from a friend or relative for free
I bought them from a friend or relative
I traded for them with a friend or relative
I took them from a friend or relative without asking
I bought them from a drug dealer or other stranger
I stole them from a doctor's office, clinic, hospital, or pharmacy
I obtained them in some other way (please explain: ______)
Don't know/prefer not to say

20. During the two weeks after surgery, did you ever take ibuprofen (Advil, Motrin) or naproxen (Aleve)?
Yes
No [if no, skip to question 22]
Don't know/prefer not to say [skip to question 22]

© 2022 Chua KP et al. *JAMA Network Open.*

21. During the first three days after surgery, did you take ibuprofen or naproxen regularly (for instance, every 6 hours on a scheduled basis) or only when you needed them?

Regularly
As needed
Don’t know/prefer not to say

22. During the two weeks after surgery, did you ever take acetaminophen (Tylenol)?

Yes
No [if no, skip to question 24]
Don’t know/prefer not to say [skip to question 24]

23. During the first three days after surgery, did you take acetaminophen regularly (for instance, every 4 hours on a scheduled basis) or only when you needed them?

Regularly
As needed
Don’t know/prefer not to say

© 2022 Chua KP et al. *JAMA Network Open.*

24. PHQ-8

How often have you been bothered by each of the following symptoms during the past **TWO WEEKS?**	Not at all	Several days	More than half the days	Nearly every day
1. Little interest or pleasure in doing things?	☐	☐	☐	☐
2. Feeling down, depressed, irritable, or hopeless?	☐	☐	☐	☐
3. Trouble falling asleep, staying asleep, or sleeping too much?	☐	☐	☐	☐
4. Feeling tired, or having little energy?	☐	☐	☐	☐
5. Poor appetite, weight loss, or overeating?	☐	☐	☐	☐
6. Feeling bad about yourself – or feeling that you are a failure, or that you have let yourself or your family down?	☐	☐	☐	☐
7. Trouble concentrating on things like school work, reading, or watching TV?	☐	☐	☐	☐
8. Moving or speaking so slowly that other people could have noticed? Or the opposite – being so fidgety or restless that you were moving around a lot more than usual?	☐	☐	☐	☐

© 2022 Chua KP et al. *JAMA Network Open.*

25. PROMIS Pediatric Anxiety – Short Form 8a

Please respond to each question or statement by marking one box per row.

	In the past 7 days…	Never	Almost Never	Sometimes	Often	Almost Always
2220R2r	I felt like something awful might happen..	☐ 1	☐ 2	☐ 3	☐ 4	☐ 5
713R1r	I felt nervous...	☐ 1	☐ 2	☐ 3	☐ 4	☐ 5
227bR1r	I felt scared	☐ 1	☐ 2	☐ 3	☐ 4	☐ 5
5044R1r	I felt worried..	☐ 1	☐ 2	☐ 3	☐ 4	☐ 5
3459bR1r	I worried when I was at home	☐ 1	☐ 2	☐ 3	☐ 4	☐ 5
2230R1r	I got scared really easy	☐ 1	☐ 2	☐ 3	☐ 4	☐ 5
231R1r	I worried about what could happen to me ..	☐ 1	☐ 2	☐ 3	☐ 4	☐ 5
3150bR2r	I worried when I went to bed at night	☐ 1	☐ 2	☐ 3	☐ 4	☐ 5

© 2022 Chua KP et al. *JAMA Network Open.*

26. PROMIS Sleep Disturbance – Short Form 4a

Please respond to each question or statement by marking one box per row.

In the past 7 days…

		Very poor	Poor	Fair	Good	Very good
Sleep109	My sleep quality was	☐ 5	☐ 4	☐ 3	☐ 2	☐ 1

In the past 7 days…

		Not at all	A little bit	Somewhat	Quite a bit	Very much
Sleep116	My sleep was refreshing.	☐ 5	☐ 4	☐ 3	☐ 2	☐ 1
Sleep20	I had a problem with my sleep.................	☐ 1	☐ 2	☐ 3	☐ 4	☐ 5
Sleep44	I had difficulty falling asleep...................	☐ 1	☐ 2	☐ 3	☐ 4	☐ 5

© 2022 Chua KP et al. *JAMA Network Open.*

27. During the two weeks after surgery, did you or one of your family members ever contact your surgeon (or one of his or her colleagues) via phone, text, or the Internet because your pain was not well-controlled?

Yes

No

28. During the two weeks after surgery, did you ever see your surgeon (or one of his or her colleagues) in an office because your pain was not well-controlled?

Yes

No

29. During the two weeks after surgery, did you or one of your family members ever contact your primary care doctor (or one of his or her colleagues) via phone, text, or the Internet because your pain was not well-controlled?

Yes

No

30. During the two weeks after surgery, did you ever see your primary care doctor (or one of his or her colleagues) in an office because your pain was not well-controlled?

Yes

No

31. During the two weeks after surgery, did you ever go to an urgent care center because your pain was not well-controlled? Urgent care centers are walk-in clinics that are often open at night and on weekends; many have the words "Urgent Care" in their name.

Yes (please specify which urgent care center: ___________)

No

32. During the two weeks after surgery, did you ever go to a retail clinic because your pain was not well-controlled? Retail clinics are walk-in clinics located inside a grocery store or pharmacy, like CVS Minute Clinics.

Yes (please specify which retail clinic: ___________)

No

33. During the two weeks after surgery, did you ever go to an emergency department at a hospital because your pain was not well-controlled?

Yes (please specify which emergency department: _______)

No

34. During the two weeks after surgery, were you ever hospitalized because your pain was not well-controlled? Please do not count emergency department visits in which you stayed overnight in the emergency department but then went home the following day.

Yes (please specify which hospital: _______)

No

© 2022 Chua KP et al. *JAMA Network Open.*

eAppendix 6. Details on the Analysis of Outcomes in the Secondary Analysis

Our study protocol listed 18 outcomes for the secondary analysis in our study protocol. For 16 of these 18 outcomes, the number of responses with missing data (including non-response or “don’t know”) ranged from 0-2; those respondents were excluded from analyses of that outcome. For the opioid consumption outcome, 15 respondents were excluded owing to missing data or data that was unintelligible (e.g., when patients typed the prescription instructions instead of the number of doses taken, such as “5 mg by mouth every 4 hours”). For the leftover opioid doses outcome, 11 respondents were excluded owing to missing data.

1) Overall satisfaction with pain control during the two weeks after surgery

This was measured using a scale of 0-10, with 0 = no pain.

2) Proportion of patients who reported that their pain was well-controlled

The alternative options were adequately controlled and poorly controlled. Per protocol, we reported the proportion of patients reporting pain was well-controlled versus adequately/poorly controlled.

3) Proportion of patients whose overall pain control was rated as much worse or worse than expected

The alternative options were about what you expected, better than you expected, and much better than expected, adequately controlled and poorly controlled. Per protocol, we reported the proportion of patients reporting that pain was worse versus much worse than expected versus the other options.

4) Proportion of patients whose pain has resolved by day 14 after surgery

This outcome was based on question 4 in the two-week post-operative survey. If patients indicated they still had pain or that pain resolved on day 14 or beyond, the outcome was coded as 0. Otherwise, the outcome was coded as 1.

5) Pain score over the past 7 days at its worst

This was measured using a scale of 0-10, with 0 = no pain.

6) Pain score over the past 7 days on average (scale of 0-10)

This was measured using a scale of 0-10, with 0 = no pain.

7) Number of opioid doses taken during the two weeks after surgery

Patients were asked to type the exact number of doses they consumed. We provided a pain diary to patients via e-mail after discharge in order to facilitate recall. If patients indicated they did not know, they were asked to estimate consumption in buckets of 5 doses (e.g., 1-5, 6-10 doses). In these instances, we took the midpoint of these values (e.g., 3 doses if 1-5 was checked).

8) Proportion of patients with leftover doses of opioids

This outcome was based on question 12 in the two-week post-operative survey. If patients indicated that they took all of the medications they were prescribed, the outcome was coded as 0 and 1 otherwise.

© 2022 Chua KP et al. *JAMA Network Open.*

9) Proportion of patients with leftover doses who disposed of them

Patients were only eligible for this question if they had leftover doses of opioids. This outcome was based on question 13 in the two-week post-operative survey.

10) Proportion of patients who misused opioids belonging to others at least once during the two weeks after surgery

This outcome was based on question 18 in the two-week post-operative survey. If subjects checked the option "At least once, I took opioid pain medications that were not prescribed to me" then the outcome was coded as 1. If they did not check this option or were not asked this question because they indicated they had not misused opioids in question 17, the outcome was coded as 0.

11) Proportion of patients who misused their own opioids at least once during the two weeks after surgery

This was based on question 18 in the two-week post-operative survey. If subjects checked any of these options, the outcome was coded as 1. If they did not check any of these options or were not asked this question because they indicated they had not misused opioids in question 17, the outcome was coded as 0.

- At least once, I took a higher amount of opioid pain medication than a doctor directed me to (for example, taking twice as much medication per dose than my doctor directed me to)
- At least once, I took opioid pain medications more often than a doctor directed me to (for example, taking the medications every two hours when my doctor directed me to take them every four hours)
- I took opioid pain medication for longer (more days) than a doctor directed me to
- I took opioid pain medication in some other way than a doctor directed me to (please explain: ______)

12) PHQ-8 score (validated depression screen)

The PHQ-8 has 8 questions. For each question, the score was 0 if the answer was "not at all", 1 if "several days", 2 if "more than half the days", 3 if "nearly every day." Scores were summed across the 8 questions to get the overall score (range 0-24).

13) Raw score on PROMIS Pediatric Anxiety – Short Form 8a

For all questions, 1 = never, 2 = almost never, 3 = sometimes, 4 = often, and 5 = almost always. We summed scores across the 8 questions to obtain the raw score (range is 8-40).

14) T-score on PROMIS Sleep Disturbance – Short Form 4a

For the first question, 1 = very good , 2 = good, 3 = fair, poor = 4, 5 = very poor. For the second question, 1 = very much, 2 = quite a bit, 3 = somewhat, 4 = a little bit, and 5 = not at all. For the third and fourth questions, 1 = not at all, 2 = a little bit, 3 = somewhat, 4 = quite a bit, 5 = very much. We summed scores across the 4 questions to obtain the raw score (range is 4-20).

15) Proportion of patients who saw their primary care provider (or a colleague) in the office due to poorly controlled pain at least once during the two weeks after surgery
16) Proportion of patients who visited an urgent care center due to poorly controlled pain at least once during the two weeks after surgery

© 2022 Chua KP et al. *JAMA Network Open.*

17) Proportion of patients who visited a retail clinic due to poorly controlled pain at least once during the two weeks after surgery
18) Proportion of patients who went to an emergency department and/or were hospitalized due to poorly controlled pain at least once during the two weeks after surgery

These outcomes were based on questions 30-34 in the post-operative day 14 survey.

© 2022 Chua KP et al. *JAMA Network Open.*

eTable 1. Characteristics of Patients in the Primary Analysis, by Treatment Group and Postintervention Status

	**Pediatric otolaryngology, overall**	**Pediatric otolaryngology, pre-intervention**	**Pediatric otolaryngology, post-intervention**	**General otolaryngology, overall**	**General otolaryngology, pre-intervention**	**General otolaryngology, post-intervention**
**Sample size**	131	70	61	106	46	60
**Surgery date**						
Oct 2019-Nov 2019	14 (10.7%)	14 (20.0%)	0 (0.0%)	5 (4.7%)	5 (10.9%)	0 (0.0%)
Dec 2019-Jan 2020	18 (13.7%)	18 (25.7%)	0 (0.0%)	16 (15.1%)	16 (34.8%)	0 (0.0%)
Feb 2020-Mar 2020	10 (7.6%)	10 (14.3%)	0 (0.0%)	6 (5.7%)	6 (13.0%)	0 (0.0%)
Jun 2020-Jul 2020	16 (12.2%)	16 (22.9%)	0 (0.0%)	10 (9.4%)	10 (21.7%)	0 (0.0%)
Aug 2020-Sep 2020	12 (9.2%)	12 (17.1%)	0 (0.0%)	9 (8.5%)	9 (19.6%)	0 (0.0%)
Oct 2020-Nov 2020	5 (3.8%)	0 (0.0%)	5 (8.2%)	6 (5.7%)	0 (0.0%)	6 (10.0%)
Dec 2020-Jan 2021	13 (9.9%)	0 (0.0%)	13 (21.3%)	15 (14.2%)	0 (0.0%)	15 (25.0%)
Feb 2021-Mar 2021	11 (8.4%)	0 (0.0%)	11 (18.0%)	11 (10.4%)	0 (0.0%)	11 (18.3%)
Apr 2021-May 2021	12 (9.2%)	0 (0.0%)	12 (19.7%)	13 (12.3%)	0 (0.0%)	13 (21.7%)
Jun 2021-Jul 2021	20 (15.3%)	0 (0.0%)	20 (32.8%)	15 (14.2%)	0 (0.0%)	15 (25.0%)
**Age group**						
12-17	119 (90.8%)	62 (88.6%)	57 (93.4%)	8 (7.5%)	3 (6.5%)	5 (8.3%)
18-25	12 (9.2%)	8 (11.4%)	4 (6.6%)	98 (92.5%)	43 (93.5%)	55 (91.7%)
**Sex**						
Male	55 (42.0%)	28 (40.0%)	27 (44.3%)	32 (30.2%)	16 (34.8%)	16 (26.7%)
Female	74 (56.5%)	40 (57.1%)	34 (55.7%)	73 (68.9%)	30 (65.2%)	43 (71.7%)
Other	2 (1.5%)	2 (2.9%)	0 (0.0%)	1 (0.9%)	0 (0.0%)	1 (1.7%)
**Race/ethnicity (self-reported)**						
White/Non-Hispanic	98 (74.8%)	54 (77.1%)	44 (72.1%)	83 (78.3%)	37 (80.4%)	46 (76.7%)
Black/Non-Hispanic	12 (9.2%)	5 (7.1%)	7 (11.5%)	9 (8.5%)	2 (4.3%)	7 (11.7%)
Hispanic, any race	9 (6.9%)	3 (4.3%)	6 (9.8%)	6 (5.7%)	3 (6.5%)	3 (5.0%)
Asian/Non-Hispanic	4 (3.1%)	4 (5.7%)	0 (0.0%)	3 (2.8%)	2 (4.3%)	1 (1.7%)
Other/multi-racial, non-Hispanic	4 (3.1%)	3 (4.3%)	1 (1.6%)	3 (2.8%)	1 (2.2%)	2 (3.3%)
Unknown race and/or ethnicity	4 (3.1%)	1 (1.4%)	3 (4.9%)	2 (1.9%)	1 (2.2%)	1 (1.7%)
**Payer type**						
Private	93 (71.0%)	47 (67.1%)	46 (75.4%)	85 (80.2%)	37 (80.4%)	48 (80.0%)
Medicaid	38 (29.0%)	23 (32.9%)	15 (24.6%)	21 (19.8%)	9 (19.6%)	12 (20.0%)
**Mental health/substance use disorders**						
Yes	63 (48.1%)	34 (48.6%)	29 (47.5%)	47 (44.3%)	18 (39.1%)	29 (48.3%)
No	68 (51.9%)	36 (51.4%)	32 (52.5%)	59 (55.7%)	28 (60.9%)	31 (51.7%)
**Indication for tonsillectomy**						
Sleep-disordered breathing, with or without tonsillitis	92 (70.2%)	48 (68.6%)	44 (72.1%)	10 (9.4%)	6 (13.0%)	4 (6.7%)
Tonsillitis without sleep-disordered breathing	32 (24.4%)	19 (27.1%)	13 (21.3%)	89 (84.0%)	37 (80.4%)	52 (86.7%)
Other	7 (5.3%)	3 (4.3%)	4 (6.6%)	7 (6.6%)	3 (6.5%)	4 (6.7%)
**Procedure type**						
Tonsillectomy alone	32 (24.4%)	15 (21.4%)	17 (27.9%)	92 (86.8%)	38 (82.6%)	54 (90.0%)

© 2022 Chua KP et al. *JAMA Network Open.*

Tonsillectomy with adenoidectomy	99 (75.6%)	55 (78.6%)	44 (72.1%)	14 (13.2%)	8 (17.4%)	6 (10.0%)
**Resident involved in surgery**						
Yes	85 (64.9%)	43 (61.4%)	42 (68.9%)	47 (44.3%)	21 (45.7%)	26 (43.3%)
No	46 (35.1%)	27 (38.6%)	19 (31.1%)	59 (55.7%)	25 (54.3%)	34 (56.7%)
**Hospitalized after surgery**						
Yes	24 (18.3%)	15 (21.4%)	9 (14.8%)	2 (1.9%)	1 (2.2%)	1 (1.7%)
No	107 (81.7%)	55 (78.6%)	52 (85.2%)	104 (98.1%)	45 (97.8%)	59 (98.3%)

© 2022 Chua KP et al. *JAMA Network Open.*

eTable 2. Characteristics of Patients in the Secondary Analysis

	Pediatric otolaryngology (n = 84)	General otolaryngology (n = 66)	P value[a]
**Surgery date**			0.36
Oct 2019-Nov 2019	7 (8.3%)	2 (3.0%)	
Dec 2019-Jan 2020	11 (13.1%)	11 (16.7%)	
Feb 2020-Mar 2020	6 (7.1%)	3 (4.5%)	
Jun 2020-Jul 2020	10 (11.9%)	5 (7.6%)	
Aug 2020-Sep 2020	10 (11.9%)	5 (7.6%)	
Oct 2020-Nov 2020	3 (3.6%)	4 (6.1%)	
Dec 2020-Jan 2021	9 (10.7%)	12 (18.2%)	
Feb 2021-Mar 2021	6 (7.1%)	9 (13.6%)	
Apr 2021-May 2021	6 (7.1%)	8 (12.1%)	
Jun 2021-Jul 2021	16 (19.0%)	7 (10.6%)	
**Age group**			<0.001
12-17	78 (92.9%)	4 (6.1%)	
18-25	6 (7.1%)	62 (93.9%)	
**Sex**			0.026
Male	36 (42.9%)	16 (24.2%)	
Female	47 (56.0%)	49 (74.2%)	
Other	1 (1.2%)	1 (1.5%)	
**Race/ethnicity (self-reported)**			0.94
White/Non-Hispanic	60 (71.4%)	51 (77.3%)	
Black/Non-Hispanic	8 (9.5%)	4 (6.1%)	
Hispanic, any race	5 (6.0%)	4 (6.1%)	
Asian/Non-Hispanic	4 (4.8%)	2 (3.0%)	
Other/multi-racial, non-Hispanic	3 (3.6%)	3 (4.5%)	
Unknown race and/or ethnicity	4 (4.8%)	2 (3.0%)	
**Payer type**			0.55
Private	65 (77.4%)	54 (81.8%)	
Medicaid	19 (22.6%)	12 (18.2%)	
**Mental health/substance use disorders**			0.41
Yes	35 (41.7%)	32 (48.5%)	
No	49 (58.3%)	34 (51.5%)	
**Indication for tonsillectomy**			<0.001
Sleep-disordered breathing, with or without tonsillitis	59 (70.2%)	5 (7.6%)	
Tonsillitis without sleep-disordered breathing	21 (25.0%)	56 (84.8%)	
Other	4 (4.8%)	5 (7.6%)	
**Procedure type**			<0.001
Tonsillectomy alone	17 (20.2%)	63 (95.5%)	
Tonsillectomy with adenoidectomy	67 (79.8%)	3 (4.5%)	
**Resident involved in surgery**			0.13
Yes	55 (65.5%)	35 (53.0%)	
No	29 (34.5%)	31 (47.0%)	

© 2022 Chua KP et al. *JAMA Network Open.*

**Hospitalized after surgery**			0.02
Yes	12 (14.3%)	2 (3.0%)	
No	72 (85.7%)	64 (97.0%)	

[a]p values derive from Fisher's exact test

© 2022 Chua KP et al. *JAMA Network Open.*

eTable 3. Number of Patients Excluded From Each Analysis in the Secondary Analysis Owing to Missing Data

**Outcome**	**# patients excluded owing to missing data**
Mean (SD) satisfaction with pain control	0
Proportion reporting well-controlled pain (vs adequately or poorly controlled)	2
Proportion reporting pain was worse or much worse than expected (vs about what was expected-better than expected, and much better than expected)	1
Proportion of patients whose pain had resolved by post-operative day 14	0
Mean (SD) pain score at its worst over the past 7 days	0
Mean (SD) pain score on average over the past 7 days	0
Mean (SD) number of opioid doses consumed during the 2 weeks after surgery	15
Proportion of patients with leftover doses of opioids	4
Proportion of patients with leftover doses who disposed of them	7
Proportion of patients reporting misuse of opioids belonging to others during the 2 weeks after surgery	3
Proportion of patients reporting misuse of their own opioids during the 2 weeks after surgery	3
Mean (SD) score on PHQ-8	2
Mean (SD) score on PROMIS Pediatric Anxiety – Short Form 8a	4
Mean (SD) score on PROMIS Sleep Disturbance – Short Form 4a	4
Proportion of patients who visited their primary care physician due to poorly controlled pain	1
Proportion of patients who visited an urgent care center due to poorly controlled pain	1
Proportion of patients who visited a retail clinic due to poorly controlled pain	1
Proportion of patients who went to an emergency department or were hospitalized due to poorly controlled pain	1

© 2022 Chua KP et al. *JAMA Network Open.*

eTable 4. Proportion of Pediatric Otolaryngology Patients in the Postintervention Period With 12 Doses in the Discharge Opioid Prescription, by Patient and Clinician Factors

	Number of patients	Number (%) with 12 doses	P value[a]
**Age group**			**0.60**
12-15	38	18 (47.4%)	
16 and older	23	9 (39.1%)	
**Sex**			**0.31**
Male	34	14 (51.9%)	
Female	27	13 (38.2%)	
**Race/ethnicity (self-reported)**			**0.35**
White/Non-Hispanic	44	22 (50.0%)	
Black/Non-Hispanic	7	2 (28.6%)	
Hispanic, any race	6	1 (16.7%)	
Asian/Non-Hispanic	0	N/A	
Other/multi-racial, non-Hispanic	1	1 (100.0%)	
Unknown race and/or ethnicity	3	1 (33.3%)	
**Payer type**			**0.38**
Private	46	22 (47.8%)	
Medicaid	15	5 (33.3%)	
**Mental health/substance use disorders**			**0.80**
Yes	32	15 (46.9%)	
No	29	12 (41.4%)	
**Prescriber type**			**0.55**
Resident	47	22 (46.8%)	
Attending	14	5 (35.7%)	
**Indication for tonsillectomy**			**0.19**
Sleep-disordered breathing, with or without tonsillitis	44	20 (45.5%)	
Tonsillitis without sleep-disordered breathing	13	7 (53.9%)	
Other	4	0 (0.0%)	
**Procedure type**			**1.00**
Tonsillectomy alone	17	8 (47.1%)	
Tonsillectomy with adenoidectomy	44	19 (43.2%)	

[a]p values are from Fisher's exact test.

© 2022 Chua KP et al. *JAMA Network Open.*
