# Supplementary material for: Association Between Default Number of Opioid Doses in Electronic Health Record Systems and Opioid Prescribing to Adolescents and Young Adults Undergoing Tonsillectomy
Source: JAMA Netw Open. 2022 Jun 30;5(6):e2219701. doi: 10.1001/jamanetworkopen.2022.19701 (PMC9247741; doi:10.1001/jamanetworkopen.2022.19701)
Supplement: Supplement 3. — Data Sharing Statement [file jamanetwopen-e2219701-s00.pdf]

Data Sharing Statement

Chua. Association Between Default Number of Opioid Doses in Electronic Health Record Systems and Opioid Prescribing to Adolescents and Young Adults Undergoing Tonsillectomy. *JAMA Netw Open*. Published June 30, 2022. doi:10.1001/jamanetworkopen.2022.19701

Data
Data available: No

Additional Information
Explanation for why data not available: We are continuing to analyze the data for other studies.
